# Orthogonal Light‐ and Enzyme‐Triggered and‐Gate Prodrugs for Senolytic Therapy

**DOI:** 10.1002/advs.78139

**Published:** 2026-10-04

**Authors:** Mengyang Chang, Kaixue Zhang, Junwei Wang, Fangchao Bi, Zhuoxin Dai, Miao Yang, Ming Liu, Huanzhi Peng, Franklin Zhang, Yuan Guo, Jigang Dai, Quanxing Liu, Wei Wang

**Affiliations:** ^1^ School of Chemistry and Chemical Biology Eastern Institute of Technology Ningbo Zhejiang People's Republic of China; ^2^ Department of Thoracic Surgery Xinqiao Hospital Army (Third Military) Medical University Chongqing People's Republic of China; ^3^ Department of Pharmacology and Toxicology The University of Arizona Tucson Arizona USA; ^4^ School of Chemical Engineering Northwest University Xi'an Shaanxi People's Republic of China

**Keywords:** cancer research, cytotoxicity, doxorubicin, fluorophore, metastasis, organoid, prodrug, senescence, taxonomy, tumor progression

## Abstract

Senescence‐associated β‐galactosidase (SA‐β‐gal) is widely used to target senescent cells, but its activity in non‐senescent tissues can limit selectivity. Here, we developed a light–enzyme AND‐gate strategy in which an o‐nitrobenzyl photocage blocks β‐galactoside cleavage until 390 nm irradiation. The strategy was evaluated using a fluorescent probe (LS‐C), a doxorubicin prodrug (LS‐D), and an ARV‐771‐based PROTAC (LS‐A). LS‐C exhibited dual‐trigger‐dependent fluorescence activation, while LS‐D preferentially induced apoptosis and LS‐A promoted BRD4 degradation in senescent cells following light activation. Across multiple senescence models, LS‐D and LS‐A showed higher senolytic indices than their corresponding parent compounds. In senescent lung cancer patient‐derived organoids, LS‐D combined with light increased organoid death, whereas either treatment alone had minimal effects. These results provide proof‐of‐concept evidence for sequential light–enzyme AND‐gating as a strategy for controlling payload activation in senescent cells under the tested in vitro and ex vivo conditions.

## Graphical abstract

An orthogonal AND‐gate prodrug platform combines 390 nm near‐UV photocleavage with SA‐β‐gal activation to control payload release in senescent cells. Its modular architecture enables 4‐MU fluorescence, doxorubicin‐mediated cytotoxicity, and ARV‐771‐driven BRD4 degradation, supporting stimulus‐controlled senolytic design in cellular and organoid models.

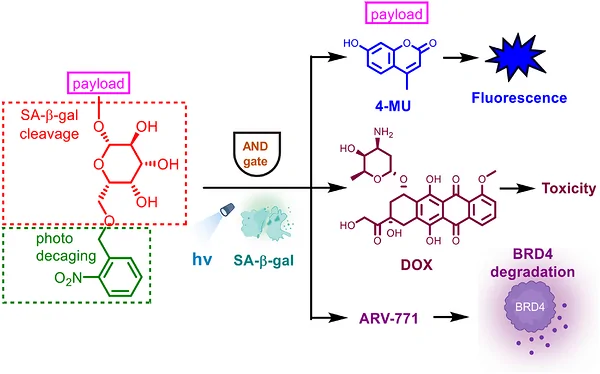

## 1 Introduction

Cellular senescence—a state of stable, irreversible cell‐cycle arrest—has emerged as a critical, yet therapeutically underexploited, hallmark of the tumor microenvironment (TME) [1, 2, 3, 4]. Therapy‐induced senescence (TIS), frequently triggered by frontline cytotoxic chemotherapy and radiotherapy, drives the accumulation of senescent cells that persist within the TME rather than undergoing clearance [5]. These persistent populations acquire a complex senescence‐associated secretory phenotypes (SASPs), characterized by the pro‐inflammatory secretome that promotes tumor recurrence, metabolic remodeling, and therapeutic resistance [6, 7]. Consequently, the selective elimination of these cells—an approach termed senolytics—is a burgeoning strategy to enhance oncological outcomes [7, 8, 9]. Despite the preclinical promise of agents such as navitoclax or the dasatinib/quercetin combination, existing senolytics are plagued by poor cell‐type selectivity. Their systemic activity leads to the depletion of physiologically essential senescent populations in healthy tissues, severely narrowing the therapeutic window and complicating clinical translation [10, 11, 12]. A chemical strategy capable of confining senolytic activity with both enhanced spatial and senescence‐associated selectivity is, therefore, a critical necessity.

Senescence‐associated β‐galactosidase (SA‐β‐gal), a lysosomal hydrolase encoded by the GLB1 gene, remains the most well‐validated and biochemically exploitable biomarker of the senescent state [13, 14, 15, 16]. Its high activity at pH 6.0 and substrate specificity for β‐galactosidic bonds have been harnessed to develop galactose‐caged prodrugs and imaging probes (Scheme 1A) [14, 17, 18, 19, 20, 21, 22, 23, 24, 25, 26, 27, 28]. However, the utility of SA‐β‐gal as a standalone trigger is increasingly scrutinized; high lysosomal activity is not exclusive to senescence [15, 29] and is observed in macrophages and highly metabolic tissues, leading to “leaky” or off‐target drug activation [30]. Although SA‐β‐gal remains a widely used marker, these limitations highlight the need to pair it with more specific biomarkers to reduce false‐positive outcomes. In addition, enzyme‐responsive prodrugs inherently lack spatial selectivity, as activation occurs wherever endogenous β‐gal activity is present. This non‐specificity limits precise control over drug release and hinders site‐selective intervention within the tumor microenvironment (TME) [31].

**SCHEME 1:**
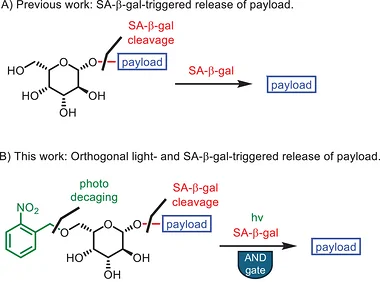
Design of SA‐β‐gal‐sensitive probes for targeting senescent cells.

In parallel, photocaged prodrugs offer a powerful paradigm for spatiotemporally controlled drug release, utilizing photolabile protecting groups to mask pharmacological activity until triggered by light [32, 33, 34, 35, 36]. Yet, photocaged systems suffer from an intrinsic lack of biological selectivity: photoactivation is agnostic to cell type, liberating payloads in all illuminated cells regardless of their physiological status [37, 38, 39]. In the heterogeneous TME, where senescent and proliferating cells exist in intimate proximity, this lack of discrimination risks collateral damage to healthy or non‐senescent populations [40]. Thus, neither enzymatic nor photochemical triggers alone satisfy the dual requirements of senolytic therapy. In our design, removal of the o‐nitrobenzyl photocage exposes the β‐galactoside substrate, making light exposure a prerequisite for subsequent SA‐β‐gal‐mediated cleavage and payload release. A distinguishing feature of the present proof‐of‐concept study is the implementation of this sequential light–enzyme AND‐gate architecture across three distinct payload classes (4‐MU, doxorubicin, and ARV‐771). Thus, our findings demonstrate that this molecular gating principle is compatible with fluorescence activation, conventional drug release, and targeted protein degradation under the experimental conditions examined [41], while Li et al. evaluated the β‐galactosidase‐targeted senolytic prodrug SSK1 in preclinical osteoarthritis models [42]. More recently, Huang et al. combined SA‐β‐gal‐initiated chemical anchoring with NIR‐mediated activation to enhance immunotherapy in senescent tumors [43]. Together with recent dual‐locked imaging, photoactivatable senolysis, and trilocked photodynamic approaches, these studies demonstrate growing interest in improving senescence selectivity through enzyme‐responsive and externally controlled systems. In comparison, the present platform directly integrates photocage removal and subsequent SA‐β‐gal cleavage within a single molecular architecture and applies this sequential AND‐gate design to three distinct payload classes, including a fluorescent reporter, a cytotoxic drug, and a PROTAC.

We reasoned that integrating these two orthogonal mechanisms into a single molecular architecture would impose a stringent AND‐gate logic on drug release (Scheme 1B) [44, 45]. In this framework, the simultaneous presence of localized irradiation and elevated SA‐β‐gal activity is required to liberate the active payload, that uniquely co‐occurs only at the illuminated senescent tumor site. This dual‐trigger design represents a conceptual leap over single‐stimulus strategies: photocleavage and enzymatic hydrolysis act as independent, mutually reinforcing gatekeepers.

Herein, we report the design, synthesis, and biological validation of a series of dual‐activated compounds incorporating a coumarin‐based fluorophore (LS‐C), the cytotoxic agent doxorubicin (LS‐D), and the proteolysis targeting chimera (PROTAC) ARV‐771 (LS‐A) as representative payloads for diagnostic and therapeutic applications. Mechanistic studies confirm the AND‐gate activation logic, validating that neither light nor enzyme alone is sufficient for payload release. Across multiple senescence models, these compounds demonstrate superior senolytic indices and precise spatiotemporal control. This work establishes a stimulus‐orthogonal framework for intervention in the TME, with potential implications for the next generation of smart prodrug systems.

## 2 Results and Discussion

### 2.1 Design, Synthesis, and Evaluation of a Light‐Controlled, SA‐β‐Gal Sensitive Coumarin‐Based Imaging Probe

Our initial study focused on designing an AND‐gate logic probe based on the widely used coumarin scaffold [46] as a fluorescent imaging tag to validate this concept. Guided by molecular docking studies, we developed a light‐controlled, senescence‐responsive fluorescent probe (LS‐C) (Figure 1A). To provide a structural rationale for the molecular design, 6‐(2‐nitrobenzyl)‐β‐galactose and β‐galactose were docked into the catalytic site of β‐galactosidase (PDB ID: 1ZJ8) using the same computational protocol. The photocaged ligand exhibited a less favorable docking score than β‐galactose (−5.637 versus −7.309 kcal mol^−^^1^; Figure 1B), supporting the hypothesis that modification of the 6‐hydroxyl group may interfere with substrate accommodation or recognition. Together with the experimental fluorescence and chromatographic results, the docking analysis provides complementary support for the proposed sequential activation mechanism. Based on this insight, a widely used photo‐cleavable *o*‐nitrobenzyl caging group [47] was introduced at the 6‐hydroxyl position via an ether linkage. It is expected that this modification effectively blocks SA‐β‐gal‐mediated cleavage of the β‐galactoside bond, thereby preventing the release of fluorescent 4‐methylumbelliferone (4‐MU). As a result, fluorescence activation is achieved only through a sequential process involving initial photocleavage followed by SA‐β‐gal‐triggered galactose hydrolysis, thereby implementing AND‐gate logic in the sensing response.

**FIGURE 1:**
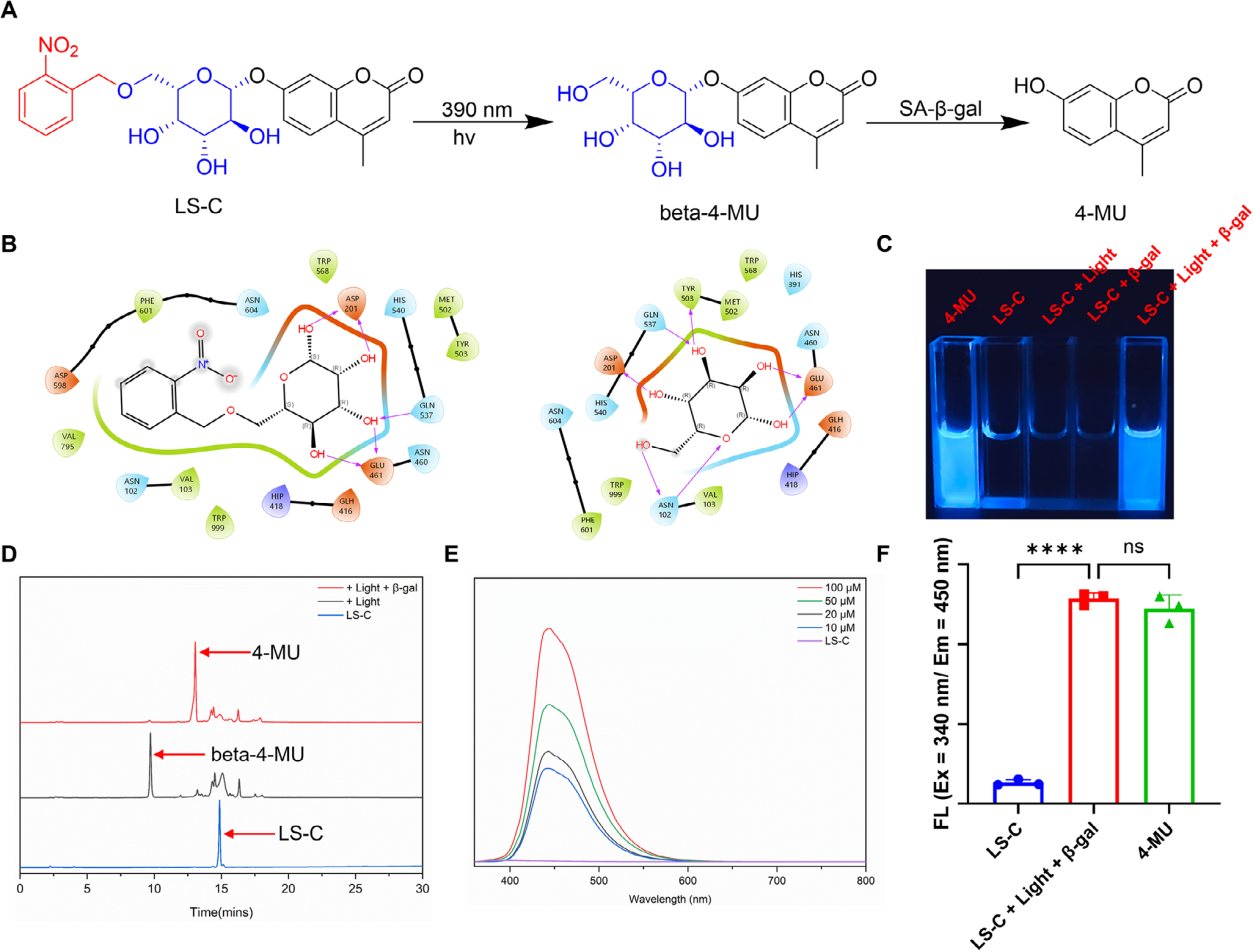
(A) Schematic illustration of the dual‐trigger mechanism, in which 390 nm ultraviolet (UV) irradiation and senescence‐associated β‐galactosidase (SA‐β‐gal) cooperatively induce the release of 4‐MU from probe LS‐C. (B) Predicted binding poses of 6‐(2‐nitrobenzyl)‐β‐galactose and β‐galactose with β‐galactosidase (PDB ID: 1ZJ8). Docking scores were calculated using Schrödinger software. The purple arrows indicate key interactions between the ligand and the protein binding pocket. (C) Fluorescence images (λ_ex_ = 340 nm) of different treatment groups (from left to right): 4‐MU (1 mm); LS‐C (1 mm); LS‐C (1 mm) pretreated with 390 nm near‐UV light for 5 min; LS‐C (1 mm) pretreated with β‐gal (2 mg) for 30 min; LS‐C (1 mm) pretreated with both 390 nm near‐UV light (5 min) and β‐gal (2 mg, 30 min). (D) Reaction profiles of LS‐C (100 µm) under dual‐trigger conditions (390 nm near‐UV light for 5 min and β‐gal (1 mg) for 30 min) in phosphate buffer (pH 7.4, 37°C), monitored by ultrafast liquid chromatography. (E) Fluorescence intensity of LS‐C at varying concentrations following treatment with 390 nm near‐UV light (5 min) and β‐gal (1 mg, 30 min) (λ_ex_ = 340 nm). (F) Quantitative fluorescence analysis was measured by a microplate reader (λ_ex_ = 340 nm, λ_em_ = 450 nm). Groups include LS‐C (100 µm); LS‐C (T), defined as LS‐C (100 µm) treated with both 390 nm near‐UV light (5 min) and β‐gal (1 mg, 30 min); and 4‐MU (100 µm) (*n* = 3).

To validate the working hypothesis, samples were irradiated at 390 nm and treated with β‐galactosidase (β‐gal). Visual inspection revealed pronounced blue fluorescence only in samples subjected to both 390 nm irradiation and β‐gal treatment, whereas irradiation or enzyme treatment alone produced no appreciable fluorescence, consistent with the expected AND‐gate behavior (Figure 1C). HPLC analysis further supported the sequential activation mechanism: irradiation of LS‐C at 390 nm generated a stable photocleavage intermediate (beta‐4‐MU), which was subsequently converted into the final fluorescent product, 4‐MU, upon β‐gal addition, confirming the stepwise dual‐trigger release (Figure 1D). The further LC‐MS data identified the release in order from beta‐4‐MU to 4‐MU (Figure S9).

Fluorescence spectroscopic characterization showed that the activated probe exhibits a maximum emission at 440–460 nm upon excitation at 340 nm, with an intensity comparable to that of free 4‐MU (Figure S1C), while uncleaved LS‐C displayed negligible background fluorescence under identical conditions (Figure 1E,F). The significant fluorescence turn‐on response, combined with minimal background signal, highlights the suitability of LS‐C as a precise fluorescent reporter for detecting SA‐β‐gal activity in senescent cells. To determine whether the selected photoactivation condition itself affected cell viability, A549 and s‐A549 cells were exposed to 390 nm near‐UV light for 5 min in the absence of compound treatment. Under this tested condition, irradiation did not significantly reduce cell viability relative to the corresponding non‐irradiated controls (Figure S1A,B).

To evaluate the performance of LS‐C in a biologically relevant senescence model, etoposide (eto) induced senescent A549 human lung carcinoma cells (s‐A549) were used [48, 49]. Senescence induction was confirmed by SA‐β‐gal staining, which revealed markedly elevated β‐galactosidase activity in s‐A549 cells compared with untreated A549 controls, validating the model for subsequent detection studies (Figure 2A). Cytotoxicity assessment using a CCK‐8 assay demonstrated that neither LS‐C nor 4‐MU exhibited significant toxicity toward normal or senescent A549 cells at the working concentrations, indicating that the probe does not compromise cellular viability or interfere with downstream imaging and analysis (Figure 2B,C).

**FIGURE 2:**
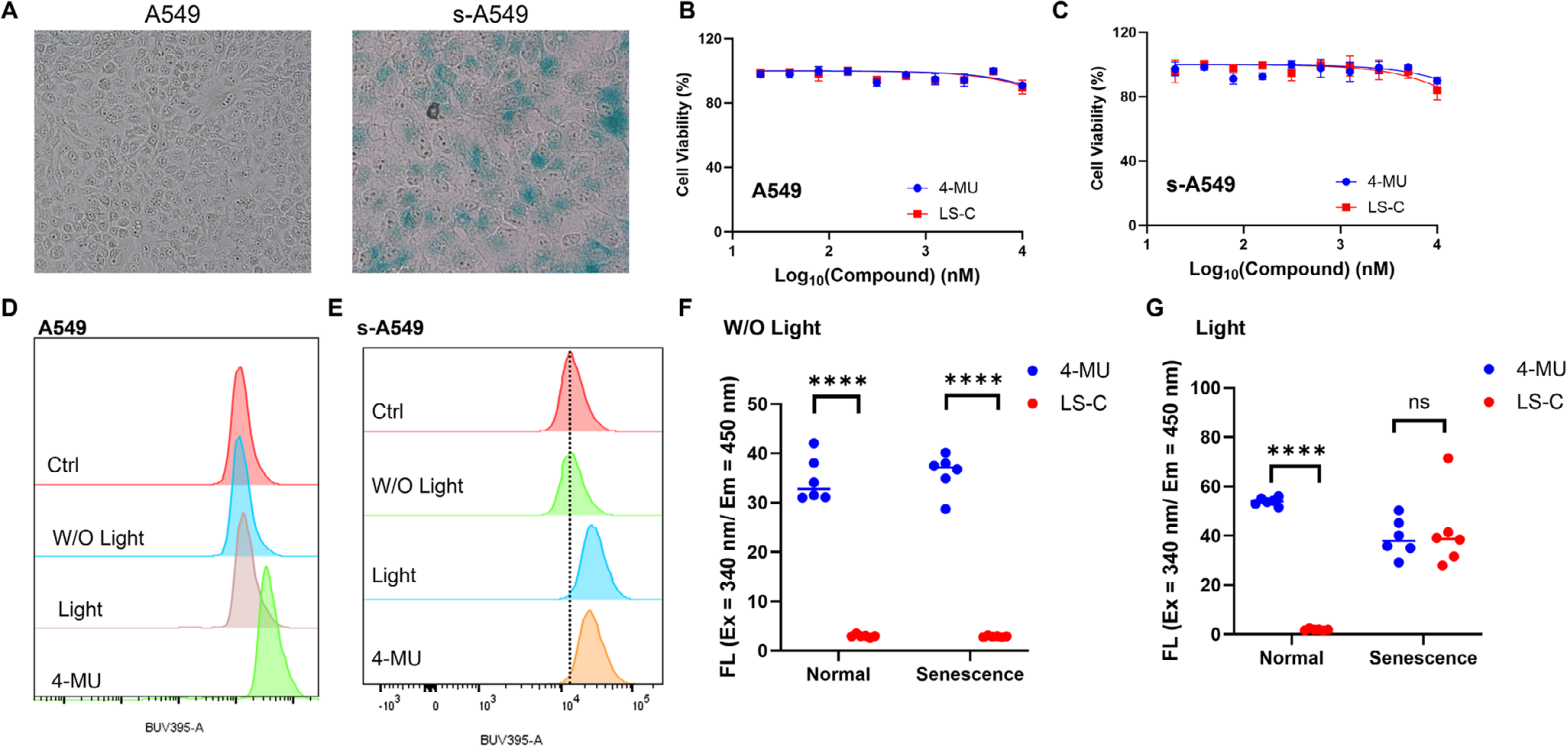
(A) Representative images of SA‐β‐gal staining in normal A549 (n‐A549) and senescent A549 (s‐A549) cells. (B, C) Cell viability of n‐A549 and s‐A549 cells following treatment with the indicated concentrations of 4‐MU or LS‐C for 72 h. (D, E) Flow cytometry analysis of LS‐C fluorescence (10 µm) in n‐A549 and s‐A549 cells, with or without 390 nm near‐UV light irradiation for 5 min. (F, G) Fluorescence intensity of 4‐MU (10 µm) or LS‐C (10 µm), measured using a microplate reader (λ_ex_ = 340 nm, λ_em_ = 450 nm).

To examine the AND‐gate activation behavior of LS‐C at the cellular level, flow cytometry was conducted in both normal and senescent A549 cells (s‐A549) with or without 390 nm irradiation. In normal A549 cells (n‐A549), where SA‐β‐gal activity is low [50, 51], LS‐C produced negligible fluorescence regardless of light exposure, consistent with the requirement for dual activation (Figure 2D). In contrast, s‐A549 cells exposed to 390 nm irradiation for 5 min exhibited fluorescence intensity comparable to that of the free 4‐MU group, confirming efficient sequential photocleavage followed by SA‐β‐gal–mediated activation in the senescent cellular environment (Figure 2E). These observations were further supported by fluorescence plate reader analysis. In the absence of 390 nm irradiation, LS‐C generated minimal fluorescence (λ_ex_ = 340 nm; λ_em_ = 450 nm) in both normal and senescent A549 cells. Upon irradiation, s‐A549 cells incubated with LS‐C displayed fluorescence intensity comparable to the 4‐MU positive control, whereas irradiated normal A549 cells maintained low background fluorescence under identical conditions (Figure 2F,G). Collectively, these results demonstrate that LS‐C functions according to AND‐gate logic in living cells, with selective fluorescence activation occurring only in senescent cells under 390 nm near‐UV light.

### 2.2 Design, Synthesis, and Evaluation of a Light‐Controlled, SA‐β‐Gal‐Sensitive Doxorubicin Prodrug

To further evaluate the light‐controlled, senescence‐targeting strategy, we extended the dual‐trigger platform to doxorubicin (Dox), a frontline anthracycline chemotherapeutic widely used in cancer therapy [52], and synthesized the corresponding photocaged prodrug, LS‐D (Figure 3A). The studies in a chemical system demonstrated that LS‐D undergoes efficient sequential activation upon 390 nm irradiation followed by β‐gal treatment, under conditions analogous to those established for LS‐C, resulting in stepwise release of the Dox payload in an AND‐gate‐dependent manner (Figure 3B, Figure S9). The senescence‐selective cytotoxicity of LS‐D was first evaluated by Western blot analysis of cleaved caspase‐3, a key marker of apoptotic cell death. In s‐A549 cells exposed to 390 nm irradiation, LS‐D induced caspase‐3 cleavage (c‐caspase‐3), whereas no activation was observed in non‐irradiated s‐A549 cells or in normal A549 cells under any condition, confirming AND‐gate‐dependent senolytic activity (Figure 3C).

**FIGURE 3:**
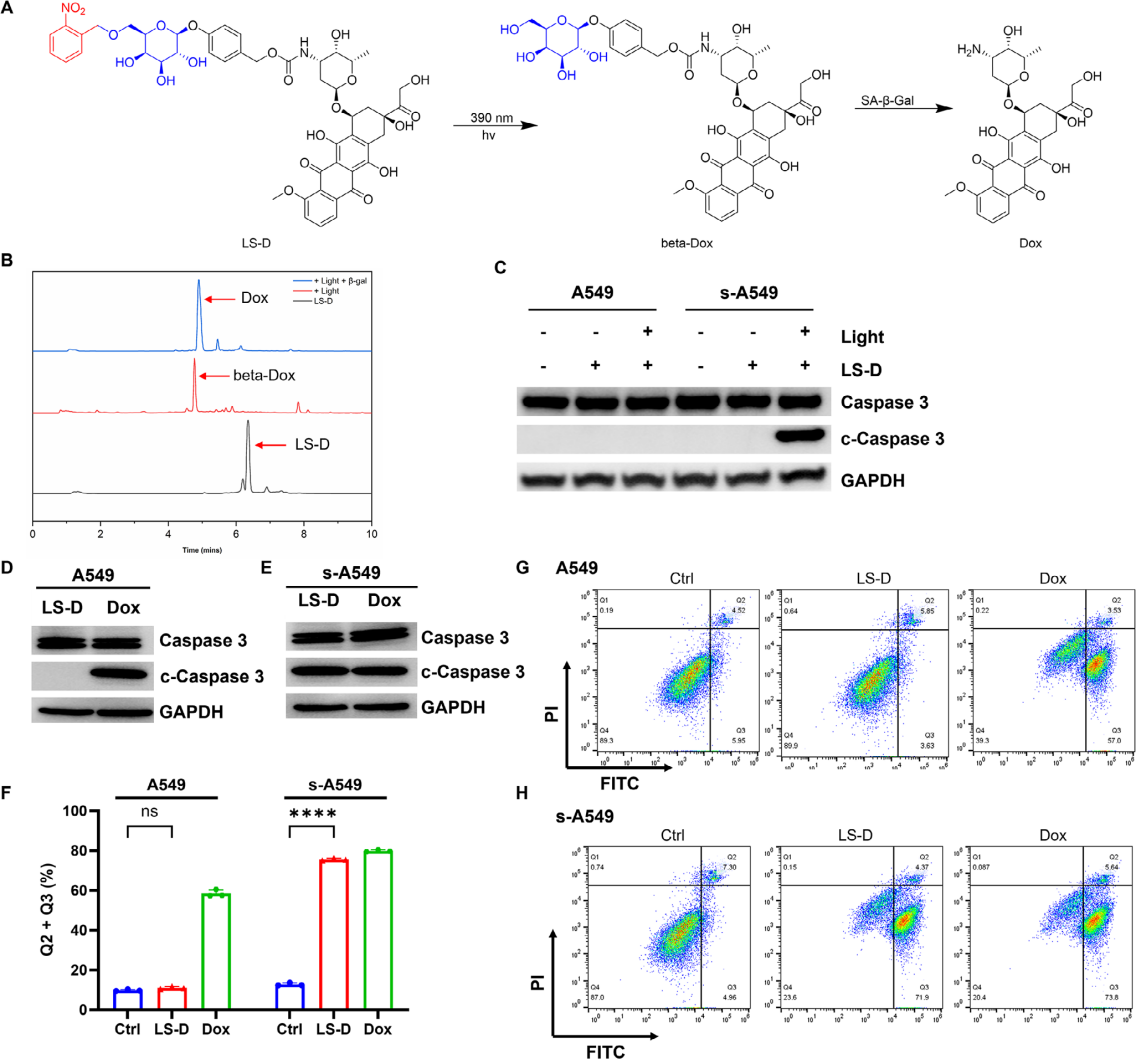
(A) Schematic illustration of dual activation of the prodrug LS‐D by 390 nm near‐UV light and SA‐β‐gal, leading to the release of doxorubicin (Dox). (B) Reaction profile of LS‐D (100 µm) following treatment with 390 nm near‐UV light (5 min) and β‐gal (1 mg, 30 min) in phosphate buffer (pH 7.4, 37°C), monitored by ultrafast liquid chromatography. (C–E) Immunoblot analysis of caspase‐3 and cleaved caspase‐3 (c‐caspase‐3) levels in A549 and s‐A549 cells. Cells were treated with 1 µm Dox or LS‐D and immediately irradiated at 390 nm for 5 min, followed by an additional 16 h of incubation. (F–H) Effects of Dox (1 µm) and LS‐D (1 µm) on apoptosis in A549 and s‐A549 cells, assessed by flow cytometry. Cells were treated with the compounds, irradiated at 390 nm for 5 min, and incubated for 48 h prior to analysis.

To benchmark therapeutic selectivity against free Dox, comparative Western blot analysis was performed in both normal and senescent A549 cells. Free Dox induced significant caspase‐3 cleavage in normal A549 cells, consistent with its well‐known non‐selective cytotoxicity, whereas LS‐D produced negligible caspase‐3 activation under identical conditions, demonstrating effective suppression of off‐target toxicity via the dual‐trigger caging strategy (Figure 3D). In contrast, in s‐A549 cells pre‐irradiated at 390 nm, LS‐D induced caspase‐3 cleavage at levels comparable to free Dox, indicating efficient prodrug activation in the senescent cellular context (Figure 3E). These findings were further corroborated by flow cytometry‐based apoptosis analysis (Figure 3F–H). LS‐D did not induce significant apoptosis in normal A549 cells regardless of irradiation status, whereas in irradiated s‐A549 cells it elicited an apoptotic response equivalent to free Dox, as reflected by comparable Q2 + Q3 populations.

Cell viability assays further substantiated the senescence‐selective activity of LS‐D. In non‐senescent A549 cells under light irradiation, LS‐D exhibited lower cytotoxic potency (IC_50_ = 1.80 µm) than free Dox (IC_50_ = 471 nm) (Figure S3A), while pre‐irradiated LS‐D exhibited potent cytotoxicity in s‐A549 cells (IC_50_ = 639 nm), approaching that of free Dox (IC_50_ = 375 nm) (Figure S3D). Importantly, in the absence of 390 nm irradiation, LS‐D retained measurable cytotoxicity, with IC_50_ values of 5.15 µm in A549 cells and 2.81 µm in s‐A549 cells. These values were higher than those observed under light activation (1.80 µm and 639 nm, respectively), indicating that irradiation increased the cytotoxic potency of LS‐D (Figure S3B,C).

To directly examine the contribution of endogenous GLB1 to cellular prodrug activation, GLB1 was knocked down in s‐A549 cells using siRNA. RT‐qPCR and immunoblotting confirmed marked reductions in GLB1 mRNA and protein expression following GLB1 siRNA transfection (Figure S10A,B). In s‐A549 cells exposed to 390 nm near‐UV light, LS‐D markedly reduced cell viability, whereas GLB1 knockdown attenuated this effect and increased viability toward the control level (Figure S10D). Meanwhile, GLB1 knockdown alone did not significantly affect cell viability. In the absence of irradiation, LS‐D did not appreciably alter the viability of either A549 or s‐A549 cells (Figure S11E,F). In contrast, free Dox reduced cell viability independently of irradiation or GLB1 status. Collectively, under the tested conditions, combined light and SA‐β‐gal activation increased the senescence‐associated selectivity of LS‐D compared with free Dox or either trigger alone, thereby achieving selective cytotoxicity while substantially sparing non‐senescent cell populations.

### 2.3 Design, Synthesis, and Evaluation of a Light‐Controlled, SA‐β‐Gal‐Sensitive ARV‐771 PROTAC

Encouraged by the demonstration of AND‐gate‐controlled senolytic activity with LS‐D, we next examined whether the dual‐trigger strategy could be extended to a proteolysis‐targeting chimera (PROTAC) therapeutic payload. PROTACs represent a mechanistically distinct therapeutic modality that induces targeted protein degradation by simultaneously engaging a protein of interest and an E3 ubiquitin ligase, thereby co‐opting the cellular ubiquitin–proteasome system (UPS) for catalytic target elimination [53, 54].

ARV‐771, a potent VHL‐recruiting PROTAC that selectively degrades bromodomain‐containing protein 4 (BRD4) [55], was chosen as the payload to design and generate the photocaged prodrug LS‐A (Figure 4A). Chemical activation studies confirmed that LS‐A undergoes efficient sequential activation upon 390 nm irradiation followed by β‐gal treatment, yielding the active BRD4 degrader ARV‐771 in a stepwise manner (Figures S4A and S9). The senescence‐selective BRD4 degradation activity of LS‐A was evaluated by Western blot analysis. In normal A549 cells, LS‐A did not induce detectable BRD4 degradation regardless of irradiation, whereas free ARV‐771 efficiently degraded BRD4 in both normal and senescent cells, consistent with its non‐selective activity (Figure 4B). In contrast, in s‐A549 cells exposed to 390 nm irradiation for 5 min, LS‐A induced BRD4 degradation to a level comparable to that of free ARV‐771, indicating complete prodrug activation and restoration of PROTAC function under dual‐trigger conditions (Figure 4C).

**FIGURE 4:**
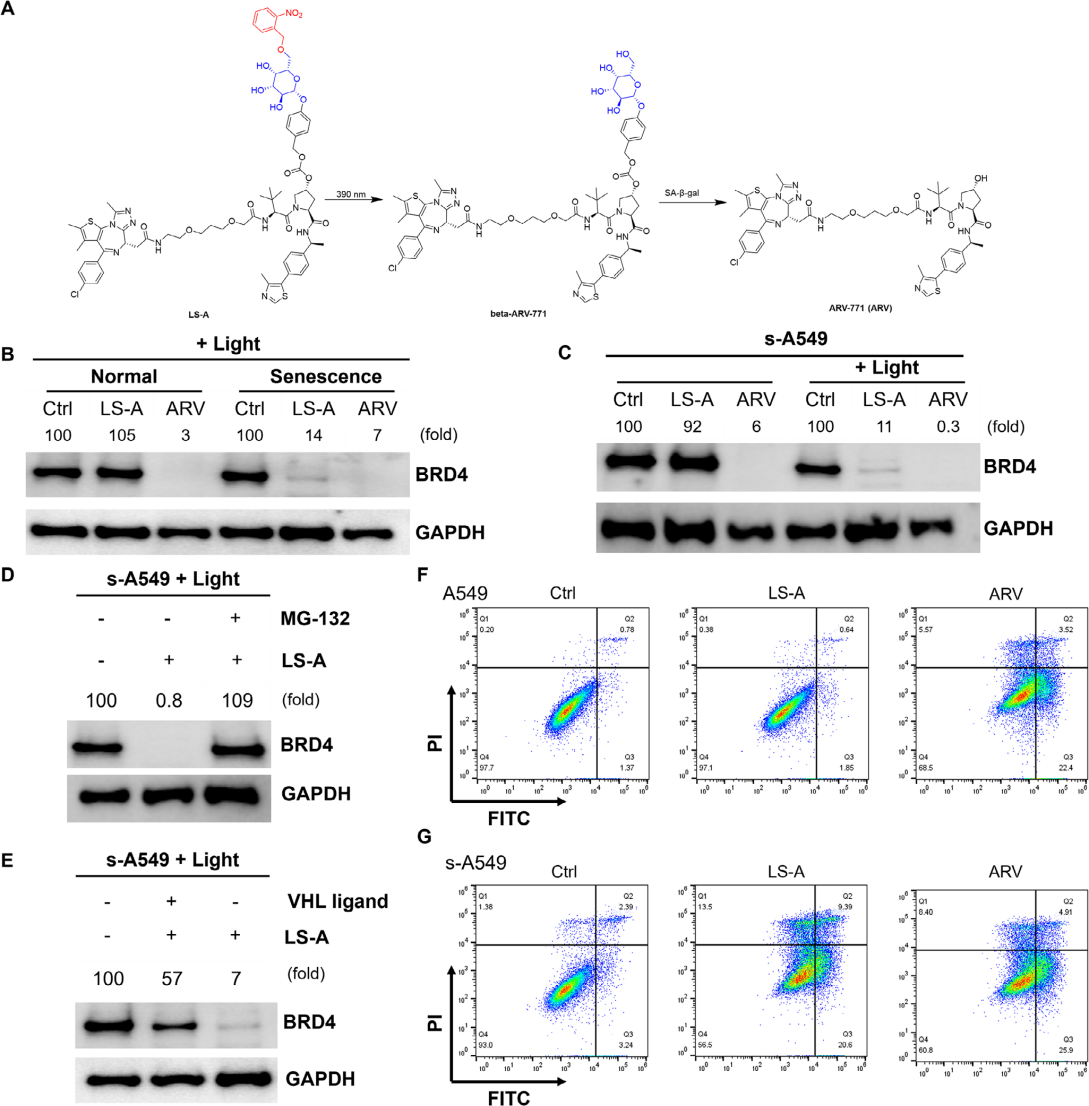
(A) Schematic illustration of dual activation of the prodrug LS‐A by 390 nm near‐UV light and SA‐β‐gal, triggering the release of ARV‐771 (ARV). (B, C) Immunoblot analysis of BRD4 and GAPDH levels in A549 and s‐A549. Cells were treated with 100 nm ARV or LS‐A and immediately irradiated at 390 nm for 5 min, and 16 h of incubation. (D, E) Immunoblot analysis of BRD4 and GAPDH levels in s‐A549 cells. Cells were pretreated with 5 µm MG‐132 or 10 µm VHL ligand, followed by treatment with 100 nM LS‐A irradiated at 390 nm for 5 min, followed by 16 h of cell treatment. (F, G) Effects of ARV (100 nm) and LS‐A (100 nm, pre‐irradiated at 390 nm) on apoptosis in A549 and s‐A549 cells, assessed by flow cytometry after 24 h.

To further elucidate the mechanism underlying LS‐A‐mediated BRD4 degradation, s‐A549 cells were pretreated with the proteasome inhibitor MG‐132 or an excess of free VHL ligand prior to LS‐A administration. Western blot analysis showed that both MG‐132 and the competitive VHL ligand abrogated BRD4 degradation, confirming that the effect proceeds via canonical E3 ligase recruitment and a ubiquitin–proteasome‐dependent pathway (Figure 4D,E). The functional consequences of BRD4 degradation were assessed by flow cytometry‐based apoptosis analysis. In normal A549 cells, LS‐A combined with 390 nm irradiation induced minimal early apoptosis (1.85%), in stark contrast to free ARV‐771 (22.4%), demonstrating effective suppression of off‐target pro‐apoptotic activity in non‐senescent cells (Figure 4F). In s‐A549 cells pre‐irradiated at 390 nm, LS‐A induced early apoptosis in 20.6% of the cell population, confirming efficient senolytic activity following dual‐trigger activation (Figure 4G). Taken together, these results demonstrate that LS‐A precisely restricts BRD4 degradation and its downstream pro‐apoptotic effects to irradiated senescent cells, further validating the AND‐gate dual‐trigger platform as a strategy for senescence‐selective PROTAC delivery.

### 2.4 Evaluation of the Senolytic Activity of LS‐D Across Multiple Senescent Cell Models and Senescence‐Inducing Conditions

To determine whether the activity of the dual‐trigger compounds depended on a particular senescence‐induction protocol, we evaluated LS‐D and LS‐A in models established using three mechanistically distinct stimuli: etoposide‐induced DNA damage, H_2_O_2_‐induced oxidative stress, and heat stress. The stability of LS‐C, LS‐D, and LS‐A was evaluated in PBS and 10% serum over 24 h (Figure S11).

Senescence was first successfully induced in both cell lines by etoposide (Eto) treatment, as confirmed by SA‐β‐gal staining (Figure S5A). Senolytic selectivity was quantified using the senolytic index (SI), defined as the ratio of IC_50_ in normal cells to IC_50_ in senescent cells, where SI > 1 indicates preferential cytotoxicity toward senescent cells. In the etoposide‐induced senescent MCF‐7 model (s‐MCF‐7), free Dox exhibited an SI of 0.514 (IC_50_: 350.9 nm in MCF‐7; 683.2 nm in s‐MCF‐7) (Figure 5A), indicating greater toxicity toward normal cells and a lack of intrinsic senolytic selectivity. In contrast, LS‐D with 390 nm pre‐irradiation achieved an SI of 8.102 (IC_50_: 3,495 nm in MCF‐7; 431.4 nm in s‐MCF‐7) (Figure 5B), representing more than a 15‐fold improvement in selectivity over free Dox. This result demonstrates that photocaging effectively suppresses off‐target cytotoxicity in normal cells while preserving potent senolytic activity upon dual‐trigger activation.

**FIGURE 5:**
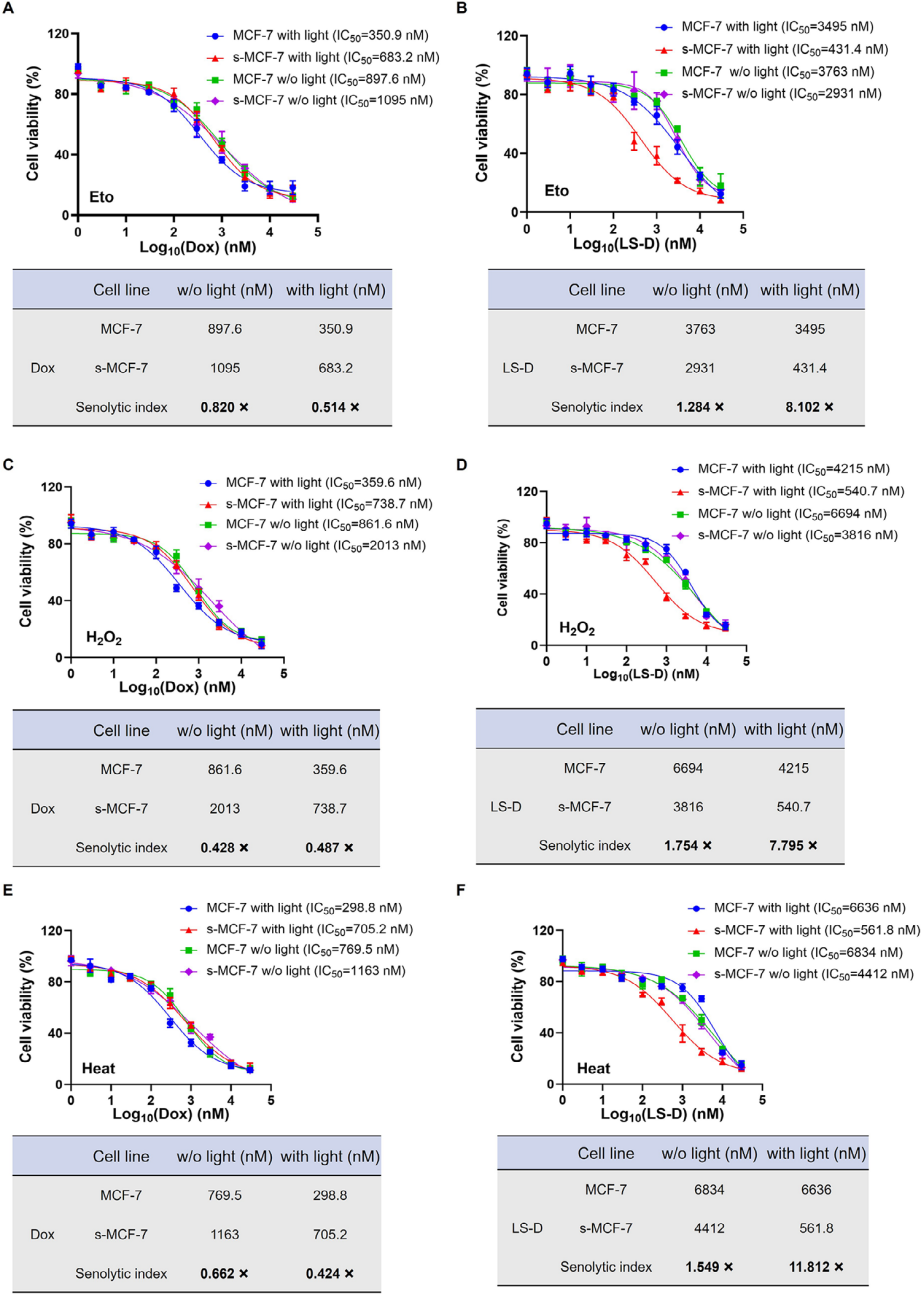
Cell toxicity of MCF‐7 and s‐MCF‐7 cells treated with indicated concentrations of Dox or LS‐D for 72 h. (A, B) ETO‐induced senescence. (C, D) H_2_O_2_‐induced senescence. (E, F) Heat‐shock‐induced senescence.

Given the dynamic and heterogeneous nature of senescence, candidate senolytic strategies should be evaluated across multiple senescent cell states that may differ in their sensitivity to existing agents [56]. To further evaluate distinct senescence‐inducing stimuli, H_2_O_2_‐ and heat shock‐induced senescent MCF‐7 models were also examined. In the H_2_O_2_‐induced model, LS‐D achieved an SI of 7.795 compared to 0.487 for free Dox (Figure 5C,D). In the heat shock‐induced model, LS‐D exhibited an SI of 11.812, whereas free Dox showed an SI of 0.662 (Figure 5E,F). Across all three MCF‐7 senescence models, LS‐D consistently demonstrated strong senolytic selectivity (SI > 7), while free Dox remained non‐selective (SI < 1).

Consistent trends were observed in the MV‐4‐11 leukemia model. In etoposide‐induced senescent MV‐4‐11 cells (s‐MV‐4‐11) with 390 nm pre‐irradiation, LS‐D achieved an SI of 4.963 (IC_50_: 2,309 nm in MV‐4‐11; 465.2 nm in s‐MV‐4‐11), substantially higher than that of free Dox (SI = 0.706; IC_50_: 329.3 nm in MV‐4‐11; 466.2 nm in s‐MV‐4‐11) (Figure S5). Collectively, these findings demonstrate that photoactivated LS‐D confers senolytic selectivity across multiple cancer cell lines and diverse senescence‐induction pathways, consistently outperforming free Dox in senolytic index.

### 2.5 Evaluation of the Senolytic Activity of LS‐A Across Multiple Senescent Cell Models

Having established the senolytic selectivity of LS‐D across multiple cancer cell lines and senescence induction models, we next evaluated the AND‐gate dual‐trigger platform using the PROTAC‐based prodrug LS‐A in etoposide‐induced senescent MCF‐7 cells (s‐MCF‐7). Consistent with observations in A549 cells, LS‐A induced efficient BRD4 degradation exclusively in s‐MCF‐7 cells following 390 nm pre‐irradiation, whereas no detectable degradation was observed in non‐irradiated s‐MCF‐7 cells or in normal MCF‐7 cells (Figure 6A–D). In contrast, free ARV‐771 (ARV) degraded BRD4 indiscriminately in both normal and senescent MCF‐7 cells, confirming the non‐selective nature of the unmodified PROTAC. Pre‐treatment with the proteasome inhibitor MG‐132 abrogated LS‐A‐mediated BRD4 degradation in s‐MCF‐7 cells, verifying that its activity proceeds through the canonical ubiquitin–proteasome pathway. To further assess robustness across distinct senescence induction mechanisms, H_2_O_2_‐ and heat shock‐induced senescent MCF‐7 models were also examined. In both cases, LS‐A combined with 390 nm pre‐irradiation achieved senolytic indices (SI) substantially greater than those of ARV, which remained below 1 under all tested conditions, consistent with trends observed for LS‐D across multiple models (Figure S6).

**FIGURE 6:**
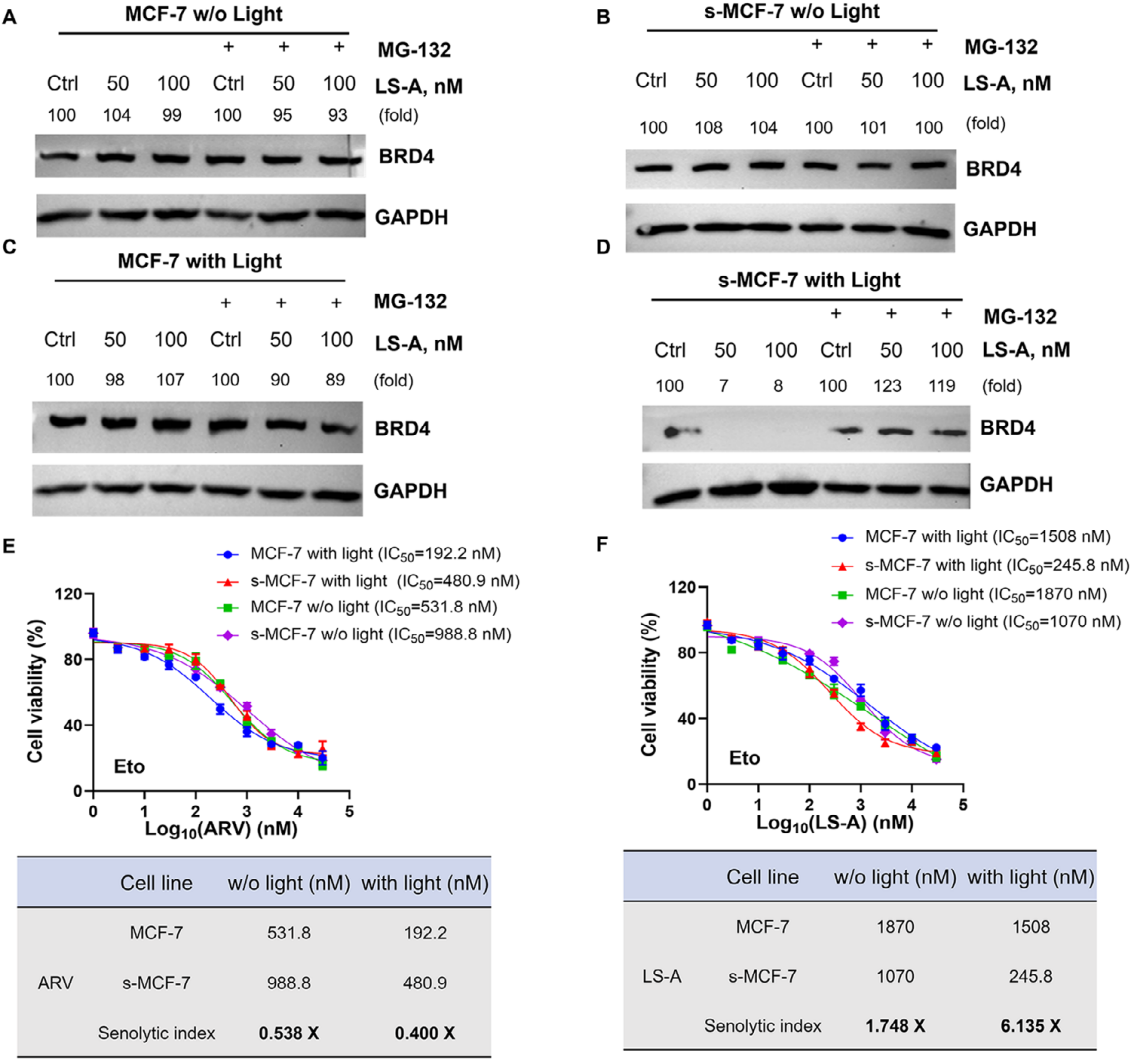
(A–D) Immunoblot analysis of BRD4 and GAPDH levels in MCF‐7 or s‐MCF‐7 cells (pre‐incubated with 5 µm MG‐132) treated with LS‐A pre‐irradiated at 390 nm for 16 h. (E,F) Cell toxicity of MCF‐7 and s‐MCF‐7 (etoposide‐induced) cells treated with indicated concentrations of ARV‐771 or LS‐A for 72 h.

Quantitative cytotoxicity profiling in etoposide‐induced s‐MCF‐7 cells further highlighted the enhanced selectivity of LS‐A. ARV exhibited an SI of 0.4 (IC_50_: 192.2 nm in MCF‐7; 480.9 nm in s‐MCF‐7), indicating greater toxicity toward normal cells and a lack of intrinsic senolytic selectivity (Figure 6E). By contrast, LS‐A with 390 nm pre‐irradiation achieved an SI of 6.135 (IC_50_: 1,508 nm in MCF‐7; 245.8 nm in s‐MCF‐7), representing an approximately 15‐fold improvement over ARV (Figure 6F). These results demonstrate that photocage installation effectively suppresses off‐target cytotoxicity in normal cells while restoring full PROTAC potency upon dual‐trigger activation. The senescence‐selective BRD4 degradation by LS‐A in the MCF‐7 model was further corroborated by Western blot analysis. Collectively, these findings establish LS‐A as a potent, senescence‐selective PROTAC degrader across multiple cancer cell lines and senescence induction pathways.

Again, various senescence‐inducing stimuli, H_2_O_2_‐ and heat shock‐induced senescent MCF‐7 models was also examined. In the H_2_O_2_‐induced model, LS‐A delivered an SI of 2.808 compared to 0.466 for free ARV‐771 (Figure S7A,B). In the heat shock‐induced model, LS‐A showed an SI of 2.951, whereas free ARV‐771 showed an SI of 0.381 (Figure S7C,D). Across all three MCF‐7 senescence models, LS‐A consistently demonstrated high senolytic selectivity while free ARV‐771 remained non‐selective.

A similar trend was observed in MV‐4‐11 cells. In etoposide‐, H_2_O_2_‐, and heat‐induced senescent MV‐4‐11 cells (Figure S8), 390 nm pre‐irradiation followed by LS‐A treatment yielded senolytic indices (SI) of 3.546, 2.318, and 7.128, respectively. These values were substantially higher than those achieved by free ARV‐771 (SI = 0.609, 0.152, and 0.238, respectively) (Figure S8). Notably, these distinct SI values highlight the pronounced heterogeneity of senescent cells generated by different induction methods. Collectively, these findings demonstrate that photoactivated LS‐A confers senolytic selectivity across multiple cancer cell lines and diverse senescence‐induction pathways, consistently outperforming free ARV‐771. Furthermore, these results underscore this AND‐gate dual‐trigger caging strategy for senolytic intervention.

### 2.6 LS‐D Executes Light‐Gated Senolysis in Senescent Lung Cancer Patient‐Derived Organoids

To test whether the AND‐gate strategy operates in a patient‐relevant three‐dimensional setting, we extended the LS‐D system from cell lines to lung cancer patient‐derived organoids (PDOs). PDOs established from resected lung tumor tissue were driven into senescence with etoposide (Figure 7A). To further characterize the senescent phenotype of the PDO model, SA‐β‐gal staining was performed in untreated and etoposide‐induced organoids. Etoposide‐treated PDOs exhibited more SA‐β‐gal‐positive staining than untreated controls (Figure 7B). Consistent with these findings, live/dead staining was performed in normal and etoposide‐induced senescent PDOs. LS‐D alone and combined LS‐D plus 390 nm light treatment did not significantly reduce viable/total area in normal PDOs. In contrast, the combined treatment significantly decreased viable/total area in senescent PDOs, accompanied by increased PI staining, whereas LS‐D alone had minimal effects (Figure 7C,D). Together, these results demonstrate that LS‐D achieves light‐ and senescence‐gated senolysis in patient‐derived lung cancer organoids, extending the AND‐gate prodrug strategy from monolayer cultures to a physiologically relevant 3D model and supporting its translational potential.

**FIGURE 7:**
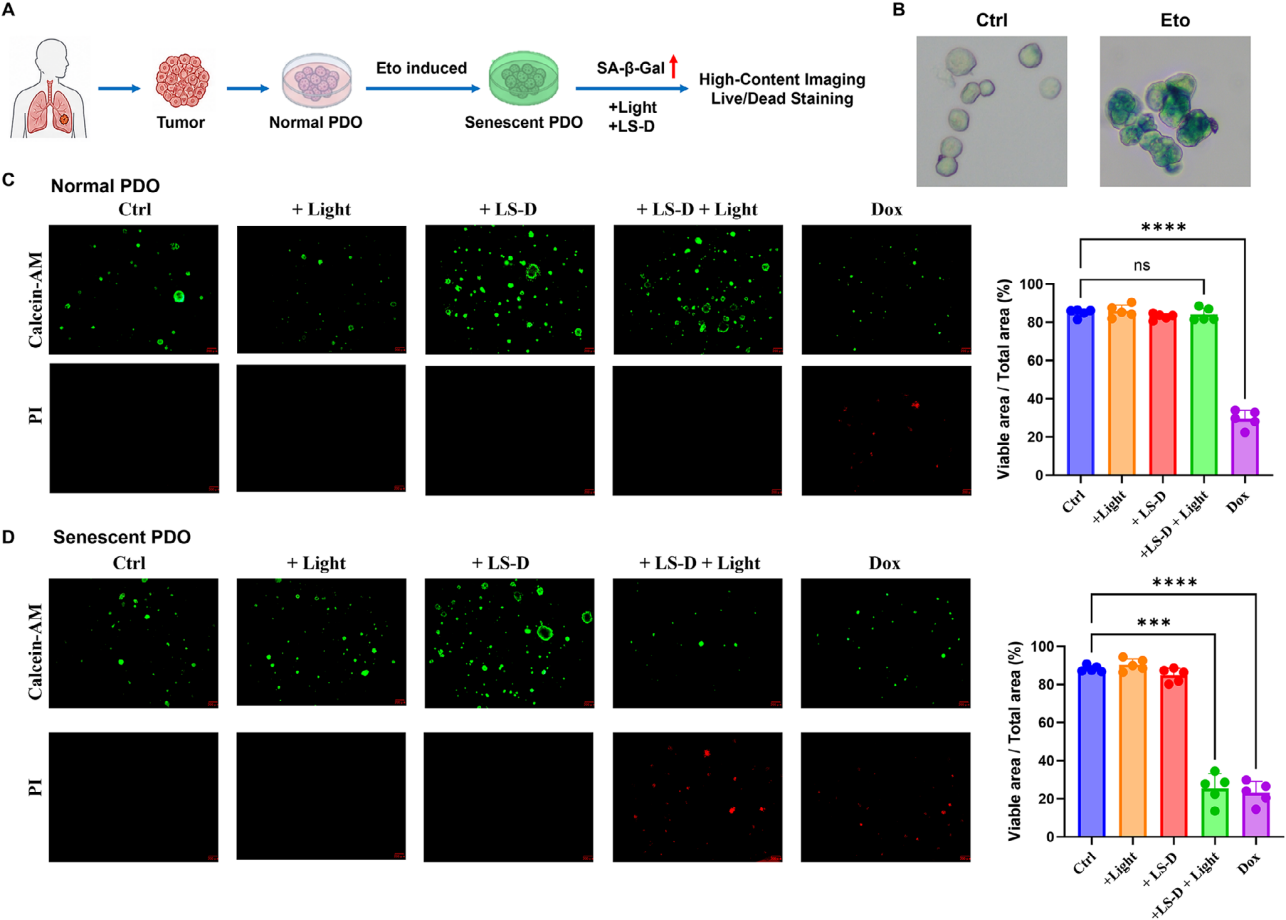
(A) Schematic of the experimental workflow. Lung cancer patient‐derived organoids (PDOs) established from tumor tissue were rendered senescent by etoposide treatment (1 µm, 5 days). (B) Representative images of SA‐β‐gal staining in untreated control (Ctrl) and etoposide‐induced (Eto, 1 µm for 5 days) PDOs. Scale bar, 50 µm. (C, D) Representative Calcein‐AM and PI staining images and quantification of viable area (Calcein‐AM positive area) and total area (Calcein‐AM positive + PI area) in normal (C) and etoposide‐induced senescent (D) PDOs after the indicated treatments (Light: 390 nm light irradiation for 5 min; LS‐D and Dox: 0.5 µm for 48 h). Data were obtained from five independent lung cancer patient donors (*n* = 5 biological replicates). Each donor was evaluated under all five treatment conditions and contributed one viable/total area value per treatment condition. PDOs from every donor were evaluated under all five treatment conditions. Statistical analysis was performed using one‐way repeated‐measures ANOVA analysis. ns, not significant; ^***^ *p* < 0.001; ^****^ *p* < 0.0001.

## 3 Discussion and Conclusion

The present findings provide proof‐of‐concept evidence that 390 nm photocleavage and SA‐β‐gal‐mediated hydrolysis can be combined to control payload activation in the experimental models examined. The AND‐gate mechanism was evaluated using three structurally and functionally distinct payloads, including a fluorescent reporter, a cytotoxic drug, and a PROTAC. Under the tested conditions, activation required both light exposure and SA‐β‐gal activity, limiting activation by either trigger alone. These results demonstrate the feasibility of the sequential dual‐trigger molecular design under the conditions examined.

SA‐β‐gal responsive prodrugs have emerged as a prominent approach for senescence‐targeted drug delivery, leveraging the elevated lysosomal β‐galactosidase activity characteristic of senescent cells to enable selective payload release in vitro [17, 18]. However, basal SA‐β‐gal activity in non‐senescent tissues may cause off‐target prodrug activation and narrow the therapeutic window. Moreover, because enzymatic activation occurs wherever substrate‐accessible enzymes are present, these systems lack spatial control, limiting their applicability in heterogeneous tumor microenvironments. In contrast, photocaged prodrugs provide precise spatiotemporal control over drug release with high photochemical efficiency and rapid kinetics [32], but inherently lack cell‐type discrimination, resulting in indiscriminate cytotoxicity across all irradiated cells. Although the light‐only controls showed minimal effects under the experimental conditions used in this study, 390 nm near‐UV light has limited tissue penetration and may cause phototoxicity at higher doses or with prolonged exposure. These limitations are likely to restrict direct application in deep‐seated tissues and therefore represent an important consideration for further development of the dual‐trigger strategy. Future studies could explore red‐shifted or near‐infrared‐responsive photocages, as well as two‐photon activation strategies, to improve effective activation depth and reduce reliance on near‐UV irradiation. Alternatively, localized fiber‐optic light delivery may provide a means to irradiate anatomically accessible internal lesions while limiting exposure of surrounding tissues. The uncaging efficiency, required light dose, tissue penetration, and safety of these approaches will need to be systematically evaluated in relevant in vivo models. This limitation is particularly acute in tumor settings, where senescent and proliferating cells coexist in close proximity. Consistent with these challenges, clinically investigated senolytics, including navitoclax and the dasatinib/quercetin combination, demonstrate that systemic administration can lead to depletion of non‐senescent cell populations and consequent dose‐limiting toxicities [10, 57, 58].

The dual‐trigger AND‐gate architecture described here addresses these limitations by requiring two independent and orthogonal activation events, such that neither stimulus alone is sufficient to induce payload release. This requirement enforces a multiplicative reduction in off‐target activation relative to single‐trigger systems. Quantitative evaluation across multiple cancer cell lines and senescence induction paradigms demonstrates the robustness of this design. Free doxorubicin and ARV‐771 consistently exhibited senolytic index (SI) values below unity, indicating preferential toxicity toward non‐senescent cells; in contrast, the corresponding dual‐trigger prodrugs reversed this selectivity profile, yielding pronounced senescence‐specific cytotoxicity. Importantly, this behavior was conserved across MCF‐7 (breast adenocarcinoma), A549 (lung carcinoma), and MV‐4‐11 (leukemia) models, indicating that the observed selectivity reflects a feature of SA‐β‐gal‐associated senescence rather than cell line effects.

We further demonstrate the modularity of this platform through its application to three structurally and functionally distinct payloads. A coumarin‐based fluorescent probe (LS‐C) serves as a mechanistic reporter and enables direct visualization of dual‐trigger activation in senescent cells. A photocaged doxorubicin prodrug (LS‐D) achieves potent senescence‐selective cytotoxicity, while a photocaged ARV‐771 PROTAC (LS‐A) enables selective degradation of BRD4, extending the dual‐trigger strategy to targeted protein degradation modalities. Together, these experiments show that the same caging principle can be applied to three distinct payload classes.

Mechanistic studies support a sequential activation pathway in which initial photocleavage unmasks the galactose substrate, enabling subsequent SA‐β‐gal‐mediated hydrolysis and release of the active payload. Relative to recently reported dual‐trigger systems, the present platform shares the general principle of requiring multiple inputs to minimize activation by either stimulus alone, but differs in how the activation logic is molecularly encoded and in the range of functional outputs. The dual‐locked bioorthogonal system reported by Wang et al. primarily supports senescence‐associated imaging, whereas the photoactivatable senolytic and trilocked photodynamic systems reported by Shi et al. and Lu et al. use light to regulate a defined senolytic or reactive oxygen species‐generating modality. Sequential chemiluminescent probes and bioorthogonally controlled photocages further illustrate how ordered activation or conditional unmasking can improve control over molecular output [27, 39]. In our design, removal of the o‐nitrobenzyl photocage exposes the β‐galactoside substrate, making light exposure a prerequisite for subsequent SA‐β‐gal‐mediated cleavage and payload release. A distinguishing feature of the present proof‐of‐concept study is the implementation of this sequential light–enzyme AND‐gate architecture across three distinct payload classes (4‐MU, doxorubicin, and ARV‐771). Thus, our findings demonstrate that this molecular gating principle is compatible with fluorescence activation, conventional drug release, and targeted protein degradation under the experimental conditions examined.

In summary, this work provides proof‐of‐concept for a modular light–enzyme dual‐trigger strategy for selective senolytic intervention across the cellular and PDO models examined in this study. By integrating spatially controlled photochemical activation with senescence‐associated enzymatic activation, this approach may help address some limitations of single‐stimulus systems and improve senolytic selectivity in the cellular and PDO models examined. Although these results establish proof‐of‐concept feasibility, several limitations must be addressed before the translational potential of this strategy can be assessed. The present study was conducted predominantly in cell‐based models, with additional validation in patient‐derived organoids, and therefore does not address in vivo pharmacokinetics, biodistribution, systemic toxicity, or the spatial control of payload activation in intact tissues. Moreover, the limited tissue penetration and potential phototoxicity of 390 nm irradiation may restrict its application to deep‐seated tissues. Future studies using relevant animal models and more tissue‐penetrant activation strategies will be required to determine whether the selectivity observed under the present experimental conditions can be maintained in vivo.

## Author Contributions

**Mengyang Chang**: writing – original draft, data curation, investigation, validation, methodology, Writing – review and editing. **Kaixue Zhang**: methodology, validation, writing – original draft, formal analysis. **Junwei Wang**: formal analysis, visualization, investigation. **Fangchao Bi**: conceptualization, investigation, formal analysis. **Zhuoxin Dai**: formal analysis, software, methodology. **Miao Yang**: methodology, data curation. **Ming Liu**: data curation, software. **Huanzhi Peng**: data curation, methodology. **Franklin Zhang**: software, formal analysis, data curation. **Yuan Guo**: writing – original draft, writing – review and editing, formal analysis, software. **Jigang Dai**: writing – original draft, writing – review and editing, supervision, funding acquisition. **Quanxing Liu**: funding acquisition, writing – original draft, writing – review and editing, project administration, supervision, resources. **Wei Wang**: resources, supervision, formal analysis, writing – review and editing, writing – original draft, funding acquisition.

## Conflicts of Interest

The authors declare no conflicts of interest.

## Supporting information




**Supporting File**: advs78139‐sup‐0001‐SuppMat.docx.

## Data Availability

The data that supports the findings of this study are available in the Supporting Information of this article.

## References

[1] V. Lucas , C. Cavadas , and C. A. Aveleira , “Cellular Senescence: From Mechanisms to Current Biomarkers and Senotherapies,” Pharmacological Reviews 75, no. 4 (2023): 675–713, 10.1124/pharmrev.122.000622. 36732079

[2] D. Muñoz‐Espín and M. Serrano , “Cellular Senescence: from Physiology to Pathology,” Nature Reviews Molecular Cell Biology 15 (2014): 482–496, 10.1038/nrm3823. 24954210

[3] M. K. Ruhland and E. Alspach , “Senescence and Immunoregulation in the Tumor Microenvironment,” Frontiers in Cell and Developmental Biology 9 (2021): 754069, 10.3389/fcell.2021.754069. PMC8529213 34692707

[4] M. Colucci , M. Sarill , and M. Maddalena , “Senescence in Cancer,” Cancer Cell 43, no. 7 (2025): 1204–1226, 10.1016/j.ccell.2025.05.015. 40513577

[5] Y. Liu , I. Lomeli , and S. J. Kron , “Therapy‐Induced Cellular Senescence: Potentiating Tumor Elimination or Driving Cancer Resistance and Recurrence?,” Cells 13 (2024): 1281, 10.3390/cells13151281. PMC11312217 39120312

[6] L. Cao , K. Li , Q. Li , Q. Tong , Y. Wang , and L. Huang , “The Controversial Role of Senescence‐associated Secretory Phenotype (SASP) in Cancer Therapy,” Molecular Cancer 24, no. 1 (2025): 283, 10.1186/s12943-025-02475-8. PMC12595836 41204284

[7] S. Chaib , T. Tchkonia , and J. L. Kirkland , “Cellular Senescence and Senolytics: the Path to the Clinic,” Nature Medicine 28, no. 8 (2022): 1556–1568, 10.1038/s41591-022-01923-y. PMC9599677 35953721

[8] T. Saliev and P. B. Singh , “Targeting Senescence: a Review of Senolytics and Senomorphics in Anti‐Aging Interventions,” Biomolecules (2025): 15, 10.3390/biom15060860. PMC12190739 40563501

[9] L. Zhang , L. E. Pitcher , V. Prahalad , L. J. Niedernhofer , and P. D. Robbins , “Recent Advances in the Discovery of Senolytics,” Mechanisms of Ageing and Development 200 (2021): 111587, 10.1016/j.mad.2021.111587. PMC8687661 34656616

[10] E. J. Novais , V. A. Tran , S. N. Johnston , et al., “Long‐term Treatment with Senolytic Drugs Dasatinib and Quercetin Ameliorates Age‐dependent Intervertebral Disc Degeneration in Mice,” Nature Communications 12, no. 1 (2021): 5213, 10.1038/s41467-021-25453-2. PMC8417260 34480023

[11] S. Maurer , V. Kirsch , L. Ruths , R. E. Brenner , and J. Riegger , “Senolytic Therapy Combining Dasatinib and Quercetin Restores the Chondrogenic Phenotype of human Osteoarthritic Chondrocytes by the Release of Pro‐Anabolic Mediators,” Aging Cell 24, no. 1 (2025): 14361, 10.1111/acel.14361. PMC11995296 39402753

[12] W. Xu , T. Zhao , H. Chen , et al., “Pan‐mTOR Inhibitors Sensitize the Senolytic Activity of Navitoclax via mTORC2 Inhibition‐mediated Apoptotic Signaling,” Biochemical Pharmacology 200 (2022): 115045, 10.1016/j.bcp.2022.115045. 35439535

[13] B. Y. Lee , J. A. Han , J. S. Im , et al., “Senescence‐Associated β‐Galactosidase Is Lysosomal β‐Galactosidase,” Aging Cell 5, no. 2 (2006): 187–195, 10.1111/j.1474-9726.2006.00199.x. 16626397

[14] J. Zhang , P. Cheng , and K. Pu , “Recent Advances of Molecular Optical Probes in Imaging of β‐galactosidase,” Bioconjugate Chemistry 30, no. 8 (2019): 2089–2101, 10.1021/acs.bioconjchem.9b00391. 31269795

[15] Y. Valieva , E. Ivanova , A. Fayzullin , A. Kurkov , and A. Igrunkova , “Senescence‐associated β‐galactosidase Detection in Pathology,” Diagnostics 12, no. 10 (2022): 2309, 10.3390/diagnostics12102309. PMC9599972 36291998

[16] F. Debacq‐Chainiaux , J. D. Erusalimsky , J. Campisi , and O. Toussaint , “Protocols to Detect Senescence‐associated Beta‐galactosidase (SA‐βgal) Activity, a Biomarker of Senescent Cells in Culture and in Vivo,” Nature Protocols 4, no. 12 (2009): 1798–1806, 10.1038/nprot.2009.191. 20010931

[17] M. Chang , Y. Dong , A. B. Cruickshank‐Taylor , G. Gnawali , F. Bi , and W. Wang , “Senolytic Prodrugs: a Promising Approach to Enhancing Senescence‐Targeting Intervention,” Chembiochem 25, no. 22 (2024): 202400355, 10.1002/cbic.202400355. PMC11576250 39058554

[18] X. Wang , Z. Huang , C. Sui , M. Xu , and X. Sun , “Advances in Small‐molecule Fluorescent Probes for Cellular Senescence Diagnosis and Therapy: a Review,” Dyes and Pigments 235 (2025): 112599, 10.1016/j.dyepig.2024.112599.

[19] D. Asanuma , M. Sakabe , M. Kamiya , et al., “Sensitive β‐galactosidase‐targeting Fluorescence Probe for Visualizing Small Peritoneal Metastatic Tumours in Vivo,” Nature Communications 6, no. 1 (2015): 6463, 10.1038/ncomms7463. PMC4382686 25765713

[20] K. Gu , Y. Xu , H. Li , et al., “Real‐time Tracking and in Vivo Visualization of β‐galactosidase Activity in Colorectal Tumor with a Ratiometric near‐infrared Fluorescent Probe,” Journal of the American Chemical Society 138, no. 16 (2016): 5334–5340, 10.1021/jacs.6b01705. 27054782

[21] B. Lozano‐Torres , I. Galiana , M. Rovira , et al., “An OFF–ON Two‐Photon Fluorescent Probe for Tracking Cell Senescence in Vivo,” Journal of the American Chemical Society 139, no. 26 (2017): 8808–8811, 10.1021/jacs.7b04985. 28625064

[22] Y. Gao , Y. Hu , Q. Liu , et al., “Two‐Dimensional Design Strategy to Construct Smart Fluorescent Probes for the Precise Tracking of Senescence,” Angewandte Chemie International Edition 60, no. 19 (2021): 10756–10765, 10.1002/anie.202101278. 33624914

[23] X. Wang , S. S. Liew , J. Huang , Y. Hu , X. Wei , and K. Pu , “Dual‐Locked Enzyme‐Activatable Bioorthogonal Fluorescence Turn‐On Imaging of Senescent Cancer Cells,” Journal of the American Chemical Society 146, no. 32 (2024): 22689–22698, 10.1021/jacs.4c07286. 39101919

[24] D. Shi , W. Liu , Y. Gao , et al., “Photoactivatable Senolysis with Single‐cell Resolution Delays Aging,” Nature Aging 3, no. 3 (2023): 297–312, 10.1038/s43587-023-00360-x. 37118423

[25] P. Lu , H. Zhou , X. Xu , Y. Long , J. H. Jiang , and F. Wang , “Trilocked Photodynamic Senolytic Inducer Potentiating Immunogenic Senescent Cell Removal for Liver Fibrosis Resolution,” Journal of the American Chemical Society 147, no. 19 (2025): 16494–16505, 10.1021/jacs.5c03498. 40305473

[26] X. Zhen , J. Zhang , J. Huang , C. Xie , Q. Miao , and K. Pu , “Macrotheranostic Probe with Disease‐Activated near‐Infrared Fluorescence, Photoacoustic, and Photothermal Signals for Imaging‐Guided Therapy,” Angewandte Chemie International Edition 57, no. 26 (2018): 7804–7808, 10.1002/anie.201803321. 29665259

[27] Y. Zhang , C. Yan , C. Wang , Z. Guo , X. Liu , and W. H. Zhu , “A Sequential Dual‐Lock Strategy for Photoactivatable Chemiluminescent Probes Enabling Bright Duplex Optical Imaging,” Angewandte Chemie International Edition 59, no. 23 (2020): 9059–9066, 10.1002/anie.202000165. 31984609

[28] Y. Cai , H. Zhou , Y. Zhu , et al., “Elimination of Senescent Cells by β‐galactosidase‐targeted Prodrug Attenuates Inflammation and Restores Physical Function in Aged Mice,” Cell Research 30, no. 7 (2020): 574–589, 10.1038/s41422-020-0314-9. PMC7184167 32341413

[29] J. A. de Mera‐Rodríguez , G. Álvarez‐Hernán , Y. Gañán , G. Martín‐Partido , J. Rodríguez‐León , and J. Francisco‐Morcillo , “Is senescence‐associated β‐galactosidase a reliable in vivo marker of cellular senescence during embryonic development?,” Frontiers in Cell and Developmental Biology 9 (2021): 623175. 10.3389/fcell.2021.623175 PMC7876289 33585480

[30] L. Zhang , L. E. Pitcher , M. J. Yousefzadeh , et al., “Cellular Senescence: a Key Therapeutic Target in Aging and Diseases,” Journal of Clinical Investigation 132 (2022): e158450, 10.1172/jci158450. PMC9337830 35912854

[31] Y. Tu , J. Gong , J. Mou , H. Jiang , H. Zhao , and J. Gao , “Strategies for the Development of Stimuli‐responsive Small Molecule Prodrugs for Cancer Treatment,” Frontiers in Pharmacology 15 (2024): 1434137, 10.3389/fphar.2024.1434137. PMC11322083 39144632

[32] P. Klan , T. Solomek , C. G. Bochet , et al., “Photoremovable Protecting Groups in Chemistry and Biology: Reaction Mechanisms and Efficacy,” Chemical Reviews 113, no. 1 (2013): 119–191, 10.1021/cr300177k. PMC3557858 23256727

[33] R. Weinstain , T. Slanina , D. Kand , and P. Klán , “Visible‐to‐NIR‐Light Activated Release: from Small Molecules to Nanomaterials,” Chemical Reviews 120, no. 24 (2020): 13135–13272, 10.1021/acs.chemrev.0c00663. PMC7833475 33125209

[34] J. M. Silva , E. Silva , and R. L. Reis , “Light‐triggered Release of Photocaged Therapeutics—Where Are We Now?,” Journal of Controlled Release 298 (2019): 154–176, 10.1016/j.jconrel.2019.02.006. 30742854

[35] H. Xiong , Y. Xu , B. Kim , et al., “Photo‐controllable Biochemistry: Exploiting the Photocages in Phototherapeutic Window,” Chem 9, no. 1 (2023): 29–64, 10.1016/j.chempr.2022.11.007.

[36] J. Li , H. Kong , C. Zhu , and Y. Zhang , “Photo‐controllable Bioorthogonal Chemistry for Spatiotemporal Control of Bio‐targets in Living Systems,” Chemical Science 11, no. 13 (2020): 3390–3396, 10.1039/C9SC06540G. PMC8152734 34109018

[37] M. Yoshimura and T. Inose , “Photocleavable Systems for Cell Biology: Conceptual Design across Molecular Modalities,” Chembiochem 26, no. 23 (2025): 202500564, 10.1002/cbic.202500564. PMC12666253 41159455

[38] G. Mazzotti , D. Hartmann , and M. J. P. Booth , “Precise, Orthogonal Remote‐Control of Cell‐Free Systems Using Photocaged Nucleic Acids,” Journal of the American Chemical Society 145, no. 17 (2023): 9481–9487, 10.1021/jacs.3c01238. PMC10161223 37074404

[39] O. Ember , K. Németh , D. Kern , A. Kormos , P. Kele , and M. Bojtár , “Double Disguise: Camouflaging Photocages for Bioorthogonally Controlled Conditional Activation,” Journal of the American Chemical Society 147, no. 42 (2025): 38889–38896, 10.1021/jacs.5c15005. PMC12550854 41085101

[40] Y. Sun , Q. Li , and J. L. Kirkland , “Targeting Senescent Cells for a Healthier Longevity: the Roadmap for an Era of Global Aging,” Life Medicine 1, no. 2 (2022): 103–119, 10.1093/lifemedi/lnac030. PMC9869767 36699942

[41] X. Gao , Y. Hu , Y. Zhang , et al., “A Galactose‐tethered Tetraphenylethene Prodrug Mediated Apoptosis of Senescent Cells for Osteoporosis Treatment,” Science Advances 11, no. 8 (2025): adr2833, 10.1126/sciadv.adr2833. PMC11838013 39970227

[42] Q. Li , T. Wu , Y. Fan , et al., “β‐galactosidase‐targeted Senolytic Prodrug Ameliorates Preclinical Models of Post‐traumatic Osteoarthritis,” EBioMedicine 122 (2025): 106015, 10.1016/j.ebiom.2025.106015. PMC12637248 41202471

[43] W. Huang , J. Chen , N. Ali , J. Tian , and W. Zhang , “Chemical Anchoring of Immunotherapeutic Drugs within Senescent Tumor Cells Overcomes Senescence‐driven Immunotherapy Resistance,” Nature Communications 17 (2026): 5048, 10.1038/s41467-026-71549-y. PMC13243604 41968151

[44] A. A. Tregubov , P. I. Nikitin , and M. P. Nikitin , “Advanced Smart Nanomaterials with Integrated Logic‐Gating and Biocomputing: Dawn of Theranostic Nanorobots,” Chemical Reviews 118, no. 20 (2018): 10294–10348, 10.1021/acs.chemrev.8b00198. 30234291

[45] L. Liu , P. Liu , L. Ga , and J. Ai , “Advances in Applications of Molecular Logic Gates,” ACS Omega 6, no. 45 (2021): 30189–30204, 10.1021/acsomega.1c02912. PMC8600522 34805654

[46] K. Szwaczko , A. Kulkowska , and A. Matwijczuk , “Advances in Coumarin Fluorescent Probes for Medical Diagnostics: a Review of Recent Developments,” Biosensors (Basel) 16 (2026): 36, 10.3390/bios16010036. PMC12839267 41590287

[47] M. P. O'Hagan , Z. Duan , F. Huang , et al., “Photocleavable Ortho‐Nitrobenzyl‐Protected DNA Architectures and Their Applications,” Chemical Reviews 123, no. 10 (2023): 6839–6887, 10.1021/acs.chemrev.3c00016. PMC10214457 37078690

[48] M. Chang , Y. Dong , H. Xu , et al., “Senolysis Enabled by Senescent Cell‐Sensitive Bioorthogonal Tetrazine Ligation,” Angewandte Chemie International Edition 63, no. 9 (2024): 202315425, 10.1002/anie.202315425. PMC11226389 38233359

[49] M. Chang , F. Gao , G. Gnawali , et al., “Selective Elimination of Senescent Cancer Cells by Galacto‐modified PROTACs,” Journal of Medicinal Chemistry 67, no. 9 (2024): 7301–7311, 10.1021/acs.jmedchem.4c00152. PMC11227109 38635879

[50] I. Duran , J. Pombo , B. Sun , et al., “Detection of Senescence Using Machine Learning Algorithms Based on Nuclear Features,” Nature Communications 15, no. 1 (2024): 1041, 10.1038/s41467-024-45421-w. PMC10838307 38310113

[51] Y. T. Chou , C. H. Hsieh , S. H. Chiou , et al., “CITED2 functions as a Molecular Switch of Cytokine‐induced Proliferation and Quiescence,” Cell Death & Differentiation 19, no. 12 (2012): 2015–2028, 10.1038/cdd.2012.91. PMC3504715 22814619

[52] A. Bisht , D. Avinash , K. K. Sahu , P. Patel , G. Das Gupta , and B. D. Kurmi , “A Comprehensive Review on Doxorubicin: Mechanisms, Toxicity, Clinical Trials, Combination Therapies and Nanoformulations in Breast Cancer,” Drug Delivery and Translational Research 15, no. 1 (2025): 102–133, 10.1007/s13346-024-01648-0. 38884850

[53] M. Békés , D. R. Langley , and C. M. Crews , “PROTAC Targeted Protein Degraders: the Past Is Prologue,” Nature Reviews Drug Discovery 21 (2022): 181–200, 10.1038/s41573-021-00371-6. PMC8765495 35042991

[54] H. Gao , X. Sun , and Y. P. T. Rao , “PROTAC Technology: Opportunities and Challenges,” ACS Medicinal Chemistry Letters 11, no. 3 (2020): 237–240, 10.1021/acsmedchemlett.9b00597. PMC7073876 32184950

[55] K. Raina , J. Lu , Y. Qian , et al., “PROTAC‐induced BET Protein Degradation as a Therapy for Castration‐resistant Prostate Cancer,” Proceedings of the National Academy of Sciences 113, no. 26 (2016): 7124–7129, 10.1073/pnas.1521738113. PMC4932933 27274052

[56] F. Neri , S. Zheng , M. A. Watson , et al., “Senescent Cell Heterogeneity and Responses to Senolytic Treatment Are Related to Cell Cycle Status during Senescence Induction,” Aging (Albany NY) 17 (2025): 2063, 10.18632/aging.206299. PMC12422821 40778874

[57] J. Chang , Y. Wang , L. Shao , et al., “Clearance of Senescent Cells by ABT263 Rejuvenates Aged Hematopoietic Stem Cells in Mice,” Nature Medicine 22, no. 1 (2016): 78–83, 10.1038/nm.4010. PMC4762215 26657143

[58] L. J. Hickson , L. G. P. Langhi Prata , S. A. Bobart , et al., “Senolytics Decrease Senescent Cells in Humans: Preliminary Report from a Clinical Trial of Dasatinib plus Quercetin in Individuals with Diabetic Kidney Disease,” EBioMedicine 47 (2019): 446–456, 10.1016/j.ebiom.2019.08.069. PMC6796530 31542391

