# Supplementary material for: Orthogonal Light‐ and Enzyme‐Triggered and‐Gate Prodrugs for Senolytic Therapy
Source: Adv Sci (Weinh). 2026 Oct 4:e78139. Online ahead of print. doi: 10.1002/advs.78139 (PMC13634484; doi:10.1002/advs.78139)
Supplement: Supplementary file 1 [file ADVS-9999-e78139-s001.docx]

***Supplementary Materials***

**Orthogonal Light- and Enzyme-Triggered AND-Gate Prodrugs for Senolytic Therapy**

Mengyang Chang,^†,‡,±^ Kaixue Zhang,^†,±^ Junwei Wang,^‡,±^ Fangchao Bi,^⋕^ Zhuoxin Dai,^‡^ Miao Yang,^‡^ Ming Liu,^‡^ Huanzhi Peng,^‡^ Franklin Zhang,^⋕^ Yuan Guo,^≠,^* Jigang Dai,^‡,^* Quanxing Liu,^‡,^* and Wei Wang^†,^*

^†^ School of Chemistry and Chemical Biology, Eastern Institute of Technology, Ningbo, Zhejiang 315200, China

^‡^ Department of Thoracic Surgery, Xinqiao Hospital, Army (Third Military) Medical University, Chongqing 400037, China

^⋕^ Department of Pharmacology and Toxicology, The University of Arizona, Tucson, Arizona 85721, United States

^≠^ Key Laboratory of Synthetic and Natural Functional Molecule Chemistry of Ministry of Education, College of Chemistry and Materials Science, Northwest University, Xi’an, Shaanxi 710127, China

^±^ M.C., K.Z., and J.W. contributed equally to this work.

*Corresponding authors: Yuan Guo: [guoyuan@nwu.edu.cn](mailto:guoyuan@nwu.edu.cn); Jigang Dai: [daijigang@tmmu.edu.cn](mailto:daijigang@tmmu.edu.cn); Quanxing Liu: [quanxing9999@tmmu.edu.cn](mailto:quanxing9999@tmmu.edu.cn); Wei Wang: [weiwang@eitech.edu.cn](mailto:weiwang@eitech.edu.cn)

**Table of Contents**

[**1.**	**Synthesis and characterization of LS-C, LS-D, LS-A**	3](#_Toc237470166)

[**2.**	**Low toxicity of 390 nm UV light irradiation alone.**	6](#_Toc237470167)

[**3.**	**s-MLE-12 cells recognize the LS-C probe.**	7](#_Toc237470168)

[**4.**	**Cell viability assay of Dox and LS-D in A549 and s-A549 cells.**	8](#_Toc237470169)

[**5.**	**LS-A induces the senescent cell death**	9](#_Toc237470170)

[**6.**	**Mutliple senescent induction cell models.**	10](#_Toc237470171)

[**7.**	**LS-D exhibits senolytic selectivity across multiple senescent cancer cells.**	11](#_Toc237470172)

[**8.**	**LS-A exhibits senolytic selectivity in senescent MCF-7 cells.**	12](#_Toc237470173)

[**9.**	**LS-A exhibits senolytic selectivity in senescent MV-4-11 cells.**	13](#_Toc237470174)

[**10.**	**LC-MS identification of LS-C, LS-D, LS-A.**	14](#_Toc237470175)

[**11.**	**GLB1 knockdown attenuates LS-D cytotoxicity in irradiated senescent A549 cells**	17](#_Toc237470176)

[**12.**	**Stability of LC-S, LS-D, LS-A**	18](#_Toc237470177)

[**13.**	**Materials**	19](#_Toc237470178)

[**14.**	**Cell culture and treatment**	19](#_Toc237470179)

[**15.**	**Immunoblot assay**	19](#_Toc237470180)

[**16.**	**Senescence induction methods**	19](#_Toc237470181)

[**17.**	**Cell viability assay**	20](#_Toc237470182)

[**18.**	**Apoptosis assay**	20](#_Toc237470183)

[**19.**	**GLB1 siRNA knockdown**	20](#_Toc237470184)

[**20.**	**PDOs culture**	20](#_Toc237470185)

[**21.**	**Calcein-AM/PI Live–Dead Staining of PDOs**	21](#_Toc237470186)

[**22.**	**^1^H and ^13^C NMR spectra**	22](#_Toc237470187)

[**23.**	**High-resolution mass spectrometry (HRMS)**	25](#_Toc237470188)

# 1. **Synthesis and characterization of LS-C, LS-D, LS-A**

**Scheme S1: General procedure for synthesis of LS-C**

Int-1: The protected sugar (500mg) was dissolved in 15mL DCM, add 33% KOH 10mL and 62mg TBAB (0.1eq), stir for 10 mins. Then add the 500mg 2-Nitrobenzyl bromide (1.2 eq), the reaction was stirred for 5 hours at RT. Separate out the organic sloven and purify using silica gel with PE/EA =10/1. LCMS show the right EM+1: 396.

Int-2: The int-1 was dissolved in TFA/H_2_O= 9/1 solution and stir for 30 mins. TLC show the finish of the reaction. Evaporate the solvent and purify using silica gel with DCM/MeOH. LCMS show the right EM+1: 316.

Int-3: The int-2 was dissolved in pyridine and add Ac_2_O (2eq), the reaction was stirred overnight at RT. Evaporate out the solvent and purify using silica gel with PE/EA= 2/1. LCMS show the right EM+1: 484.

Int-4: The int-3 100mg was dissolved in DCM and add the BiBr3 (5mg, 0.05eq), then add the TMSBr (107uL, 4eq). the reaction was stirred 2 hours at RT. Evaporate out the solvent and purify using silica gel with PE/EA= 2/1. LCMS show the right EM+1: 504.

Int-5: 60mg int-4 was dissolved in MeCN, add 55mg Ag_2_O (2eq), and 4-Methylumbelliferone (23mg, 1.1 eq). The mixture was stirred for 3 hours at RT. Diluted with EtOAc and filter out the solid, the filtrate was concentrated and purified using silica gel with PE/EA= 2/1. LC-MS find the right EM+1: 600.

TM (LS-C): The int-5 was dissolved in MeOH, and and a catalytic amount of K_2_CO_3_, the reaction was stirred for 2 hours at RT. Amount of Dowex 50WX8 resin was added into the solvent and stir for 10 min, and filter out solid, the filtrate was concentrated and purify using silica gel with DCM/MeOH= 20/1. LC-MS find the EM+1: 474. HRMS find the right EM+1: 474.1392. 1H NMR (400 MHz, DMSO-D6) δ 8.04 (d, 1H), 7.80 (d, 1H), 7.75 – 7.61 (m, 2H), 7.54 (d, 1H), 7.13 – 6.96 (m, 2H), 6.26 (d, 1H), 5.41 – 5.29 (m, 1H), 5.14 – 4.95 (m, 2H), 4.86 (d, 2H), 4.80 – 4.70 (m, 1H), 4.03 (s, 1H), 3.71 (s, 2H), 3.63 (s, 2H), 3.49 (s, 1H), 2.39 (s, 3H). 13C NMR (101 MHz, DMSO-D6) δ 160.65, 160.56, 154.93, 154.23, 147.31, 135.11, 134.39, 128.90, 128.84, 126.87, 124.98, 114.50, 113.96, 112.18, 103.47, 100.59, 74.47, 73.51, 70.84, 70.44, 69.31, 69.18, 18.67.

**Scheme S2: General procedure for synthesis of LS-D**

Int-6: The int-4 (200mg), and 4-Hydroxybenzaldehyde (48mg, 1eq) and Ag_2_O (92mg, 2eq) was dissolved in to MeCN, stir at RT for 2 hours. TLC show the reaction finished. Filter out the solid and the filtrate was concentrated and purified using silica gel with PE/EA= 2/1. LCMS show the right EM+1: 546.

Int-7: The aldehyde intermediate was dissolved in DCM/MeOH (4/1), add 5eq of NaBH_4_, stir for 30mins, monitor with TLC. The mixture was filtered and purified using silica gel with PE/EA= 2/1.LCMS show the right EM+1: 548.

Int-8: The alcohol intermediate (200mg), 4-Nitrophenyl chloroformate (1.2eq, 88mg) was dissolved in DCM at 0^o^C, add TEA (3eq, 152 uL), the reaction was stirred at RT for 1 hour. Purification using silica gel with PE/EA= 2/1. LCMS show the right EM+1: 713.

Int-9: Int-8 (50mg), DOX.HCl (1.2eq) was dissolved in DMF, add TEA (4eq), the reaction was stirred for 3 hours at RT. Add EA washed with water, the organic phase was concentrated and purified using silica gel with DCM/MeOH (20/1). LCMS show the right EM+1: 1117.

TM (LS-D): The int-8 (30mg) was dissolved in MeOH, add actletic amount of K_2_CO_3_, the reaction was stirred for 1 hour at RT. The mixture was purified using silica gel with DCM/MeOH (10/1), get red solid about 10mg. . LCMS find the right EM+1: 991. HRMS find the right EM+18: 1008.3243.

1H NMR (400 MHz, DMSO-D6) δ 13.99 (s, 1H), 13.25 (s, 1H), 7.90 (d, 3H), 7.66 (d, 2H), 7.56 (t, 1H), 7.48 (t, 1H), 7.17 (d, 2H), 6.95 (d, 2H), 6.84 (d, 1H), 5.48 (s, 1H), 5.21 (d, 2H), 4.92 (dq, 4H), 4.80 (t, 2H), 4.74 (dd, 2H), 4.67 (d, 2H), 4.58 (d, 2H), 4.17 (q, 1H), 3.97 (s, 3H), 3.85 (dd, 1H), 3.75 (s, 1H), 3.68 – 3.51 (m, 5H), 2.96 (d, 2H), 2.20 (d, 1H), 2.09 (dd, 1H), 1.83 (dd, 1H), 1.46 (d, 1H), 1.25 – 1.14 (m, 1H), 1.12 (d, 3H). 13C NMR (101 MHz, DMSO-D6) δ 214.47, 187.04, 186.94, 161.29, 157.48, 156.64, 155.88, 155.01, 147.16, 136.76, 136.05, 135.17, 134.99, 134.57, 134.27, 130.87, 129.95, 128.95, 128.79, 124.80, 120.47, 120.22, 119.49, 116.49, 111.23, 111.09, 101.03, 75.45, 73.96, 73.58, 70.77, 70.62, 70.50, 69.20, 69.06, 68.55, 67.16, 65.24, 64.22, 60.31, 57.10, 47.65, 40.61, 40.51, 40.40, 40.19, 39.98, 39.78, 39.57, 39.36, 17.56, 0.65

**Scheme S3: General procedure for synthesis of LS-A**

ARV771-carbonate: ARV771 (40mg), 4-Nitrophenyl chloroformate (33mg, 4eq) and TEA (56uL, 10eq) was added into DCM at 0^o^C, then stirred at RT for 3 hours. The reaction was concentrated and purified using silica gel with DCM/MeOH (20/1). LCMS show the right EM+1: 1151.

TM (25AP124): The carbonate intermediate (35mg), and the alcohol (22mg, 1.2 eq) and TEA (42uL, 10eq) was added DMF, the mixture was stirred at RT overnight. The reaction was diluted by EA and washed by water, the organic phase was concentrated and purified using silica gel with DCM/MeOH (10/1). LCMS find the right EM+1: 1433. HRMS find the right EM+1: 1433.5024, and EM/2 + 1: 717.2561.

1H NMR (400 MHz, CHLOROFORM-D) δ 8.72 (s, 1H), 8.02 (d, J = 8.1 Hz, 1H), 7.73 (t, J = 8.0 Hz, 2H), 7.52 (t, J = 7.6 Hz, 1H), 7.40 (d, J = 8.0 Hz, 4H), 7.32 (d, J = 9.5 Hz, 7H), 7.24 (s, 1H), 7.08 (dd, J = 25.0, 8.8 Hz, 3H), 5.22 (s, 1H), 5.16 (d, J = 11.8 Hz, 1H), 5.03 (dd, J = 16.9, 8.4 Hz, 2H), 4.89 (t, J = 8.7 Hz, 2H), 4.76 (t, J = 8.1 Hz, 1H), 4.69 (d, J = 5.9 Hz, 1H), 4.62 (d, J = 9.6 Hz, 1H), 4.41 (d, J = 12.2 Hz, 1H), 4.05 (s, 1H), 3.99 (t, J = 8.1 Hz, 1H), 3.84 (d, J = 9.9 Hz, 3H), 3.74 (s, 3H), 3.64 (s, 4H), 3.57 – 3.54 (m, 2H), 3.47 (t, J = 5.9 Hz, 4H), 2.65 (s, 3H), 2.51 (s, 3H), 2.40 (s, 3H), 2.33 – 2.28 (m, 1H), 1.85 (q, J = 3.5 Hz, 3H), 1.67 (s, 3H), 1.42 (d, J = 4.3 Hz, 4H), 1.25 (s, 4H), 1.03 (s, 9H). 13C NMR (101 MHz, CHLOROFORM-D) δ 171.45, 169.83, 169.35, 164.11, 157.62, 155.62, 154.22, 148.22, 147.16, 143.33, 137.08, 134.89, 133.86, 131.15, 130.39, 130.02, 129.56, 128.83, 128.10, 126.58, 125.62, 124.72, 117.07, 101.08, 77.45, 77.13, 76.81, 74.10, 73.79, 70.64, 70.05, 69.82, 69.50, 68.98, 68.08, 67.87, 58.14, 56.38, 54.15, 53.55, 50.92, 49.06, 39.64, 36.15, 35.52, 34.75, 34.62, 31.69, 30.39, 29.80, 29.41, 29.15, 26.50, 25.70, 25.36, 22.76, 22.17, 20.80, 18.86, 16.06, 14.52, 14.24, 13.24, 11.86, 11.55.

# 2. **Low toxicity of 390 nm UV light irradiation alone.**

**Figure S1**. (A, B) Cell viability of A549 and s-A549 cells treated with 390 nm UV light irradiation (+ Light) alone for 5 mins. (C) Fluorescence emission spectrum of free 4-MU recorded upon excitation at 340 nm.

# 3. **s-MLE-12 cells recognize the LS-C probe.**

**Fig S2.** (A) Representative images of SA-β-Gal staining between MLE-12 and s-MLE-12 groups. (B, C) Cell toxicity of MLE-12 and s-MLE-12 cells treated with indicated concentrations of 4-MU or LS-C for 72 h. (D, E) Flow cytometry profiles showing the fluorescence signal of LS-C (10 μM) with or without 390 nm light for 5 mins in MLE-12 and s-MLE-12 cells.

# 4. **Cell viability assay of Dox and LS-D in A549 and s-A549 cells.**

**Figure S3.** (A-D) Cell toxicity of A549 and s-A549 cells treated with indicated concentrations of doxorubicin or LS-D for 72 h.

# 5. **LS-A induces the senescent cell death**

**Figure S4.** (A) Reaction process of 390 nm UV light (5 mins) and β-Gal (1mg, 30 mins) treated with LS-A (10 μM, pH = 7.4 phosphate buffer at 37 °C) monitored by ultrafast liquid chromatography. (B) Immunoblot analysis of BRD4, BRD3 and BRD2 levels in A549 cells treated with ARV-771 or LS-A preirradiated at 390 nm for 16 h. (C-E) Effects of compounds ARV (100 nM), LS-A (100 nM, preirradiated at 390 nm) on the induction of flow cytometry apoptosis assay in A549 or s-A549 cells for 24 h.

# 6. **Mutliple senescent induction cell models.**

**Figure S5.** (A, C, E) Representative images of SA-β-Gal staining between MCF-7 and s-MCF-7 groups with indicated concentrations of etoposide, H_2_O_2_, and temperature. (B, D, F) Representative images of SA-β-Gal staining between MV-4-11 and s-MV-4-11 groups with indicated concentrations of etoposide, H_2_O_2_, and temperature.

# 7. **LS-D exhibits senolytic selectivity across multiple senescent cancer cells.**

**Figure S6.** (A-F) Cell toxicity of MV-4-11 and s-MV-4-11 cells treated with indicated concentrations of doxorubicin or LS-D for 72 h.

# 8. **LS-A exhibits senolytic selectivity in senescent MCF-7 cells.**

**Figure S7.** (A-D) Cell toxicity of MCF-7 and s-MCF-7 cells treated with indicated concentrations of ARV-771 or LS-A for 72 h.

# 9. **LS-A exhibits senolytic selectivity in senescent MV-4-11 cells.**

**Figure S8.** (A-F) Cell toxicity of MV-4-11 and s-MV-4-11 cells treated with indicated concentrations of ARV-771 or LS-A for 72 h.

# 10. **LC-MS identification of LS-C, LS-D, LS-A.**

beta-4-MU:

4-MU:

beta-Dox:

Dox:

beta-ARV-771:

ARV-771:

**Figure S9.** LC-MS identification of release from LS-C, LS-D, LS-A.

# 11. **GLB1 knockdown attenuates LS-D cytotoxicity in irradiated senescent A549 cells**

**Figure S10.** (A) Relative GLB1 mRNA expression in s-A549 cells transfected with non-targeting control siRNA (Ctrl) or GLB1-targeting siRNA (GLB1 siRNA), as determined by RT-qPCR. (B) Immunoblot analysis of GLB1 protein expression in Ctrl and GLB1 siRNA transfected s-A549 cells. β-Actin was used as the loading control. Each lane represents an independent biological replicate. (C, D) Viability of A549 (C) and s-A549 (D) cells treated with Ctrl, GLB1 siRNA, LS-D (0.1 μM), GLB1 siRNA plus LS-D (0.1 μM), or Dox (0.1 μM) following exposure to 390 nm near-UV light for 72 h. (E, F) Viability of A549 (E) and s-A549 (F) cells subjected to the same treatments without irradiation. LS-D and Dox were used at 0.1 μM for 72 h. Data are presented as mean ± SD (n = 3 independent experiments for A and B; n = 6 for C–F). Statistical significance was determined using One-way repeated-measures ANOVA. ****P < 0.0001; ns, not significant.

# 12. **Stability of LC-S, LS-D, LS-A**

**Figure S11.** Stability of LS-C, LS-D, and LS-A in PBS and DMEM medium with 10% serum. Representative HPLC chromatograms of LS-C (A, B), LS-D (C, D), and LS-A (E, F) following incubation in PBS (A, C, E) or 10% serum (B, D, F) for 0, 4, 8, and 24 h.

# 13. **Materials**

Common materials or chemical reagents were purchased from commercial sources and used without further purification. The solvents were used by dry solvents system. All reactions were monitored by TLC or LC-MS. Purification was conducted on preparative flash column chromatography and preparative reversed-phase high performance liquid chromatography (RP-HPLC) with solvent systems specified. Nuclear magnetic resonance (NMR) spectra were recorded on automated Bruker AVIII-500 instruments. High-resolution mass spectra (HRMS) were recorded on a Bruker microTOF II instrument in positive ion mode using an Agilent G1969 API-TOF with an electrospray ionization (ESI) source. Analytical HPLC chromatography was performed on a Shimadzu LC-20AD UFLC equipped with a 4.6 mm C18 reverse-phase column. All separations involved mobile phase of 0.1% TFA (v/v) in water (solvent A) and 0.1% TFA (v/v) in ACN (solvent B).

Anti-BRD4 antibody (catalog no. 13440S), BRD3 antibody (catalog no. 94032S) and Anti-BRD2 antibody (catalog no. 5848S) were purchased from Cell Signaling Technology. Anti-GAPDH (catalog no. M7815) and Anti-Rabbit antibodies (catalog no. [MFCD00162788](https://www.sigmaaldrich.com/US/en/search/mfcd00162788?focus=products&page=1&perPage=30&sort=relevance&term=MFCD00162788&type=mdl_number)) were purchased from Millipore Sigma. Anti-mouse antibody (catalog no. A28177) was purchased from the Thermo Fisher. Anti-Cleaved Caspase 3 (catalog no. 25128-1-AP) and Anti-Caspase 3 (catalog no. 19677-1-AP) antibody were purchased from Proteintech. All primary antibodies were used at a 1:1000 dilution in 5% non-fat milk in Phosphate-buffered saline with 0.1% Tween-20 (PBST) buffer for western blot assay. All secondary antibodies were used at a 1:1000 dilution in 5% non-fat milk in Phosphate-buffered saline with 0.1% Tween-20 (PBST) buffer for western blot assay.

Photoactivation was performed using a Kessil PR160-390 LED light source (390 nm). The manufacturer-reported irradiance was 196 mW/cm^2^ at a distance of 6 cm. In the present study, samples were positioned 30 cm from the light source and irradiated for 5 min. Based on an inverse-square approximation, the irradiance at 30 cm was estimated to be 7.84 mW/cm^2^, corresponding to a total light dose of 2.35 J/cm^2^.

# 14. **Cell culture and treatment**

HeLa cells were maintained in Dulbecco’s modified Eagle’s medium (DMEM) containing 10% FBS (GIBCO, catalog no. 10437) and 1 % penicillin/streptomycin (Thermo Fisher, catalog no.30-002-Cl). SJSA-1, MOLM-13, K562, and K562/R were maintained in RPMI 1640 Medium containing 10% FBS (GIBCO, catalog no. 10437) and 1 % penicillin/streptomycin (Thermo Fisher, catalog no.30-002-Cl). Cells grew at 37 °C with 5% CO_2_.

For the 390 nm UV subgroup, cells were pre-incubated with drug-containing medium for 5 mins, followed by UV exposure (PR160L-390 nm). Immediately after irradiation, plates were returned to the incubator for continued culture.

# 15. **Immunoblot assay**

Cells were lysed in RIPA buffer supplemented with protease inhibitors. Lysates were then resolved by 4%-12% Mini Protein Gel (Thermo Fisher) at 70 V for 10 mins and 200 V for 35 min. Then proteins were transferred from the gel to PVDF membrane (Bio-Rad) at 20 V for 120 min. The membrane was incubated with primary antibody at 4 °C overnight, washed 3 times with PBST, incubated secondary antibody in 5% nonfat milk for 60 min at room temperature and then washed 3 times with PBST. The membranes were detected under the SuperSignal West Atto Ultimate Sensitivity Chemiluminescent Substrate (catalog no. [A38555](https://www.thermofisher.com/order/catalog/product/A38555)).

# 16. **Senescence induction methods**

Six-well plates were inoculated with MCF-7 cells (1×10^5^ cells per well) and grown to ~80% confluency. Cells were exposed to etoposide (Adipogen Life Sciences) at 1, 5, or 10 μM; vehicle controls received DMSO ≤0.1% (v/v). After 24, 48, or 72 h, the drug medium was replaced with fresh DMEM, and cultures were maintained for an additional 24 h. Senescence was tested using a senescence-associated β-galactosidase (SA-β-gal) kit (Cell Signaling Technology) according to the manufacturer’s instructions. The condition of 5 μM etoposide for 48 h yielded the highest positivity while maintaining cell integrity and was therefore selected for subsequent experiments. To confirm the applicability of this optimal condition to MV4-11 cells, the same protocol was applied to MV4-11 cells exposed to 5 μM etoposide for 48 h. SA-β-gal staining confirmed that this condition effectively induced senescence in MV4-11 cells.

MCF-7 cells were plated as above and, at ~80% confluency, the original serum-containing medium was aspirated. Cells were then challenged with H_2_O_2_ (Fisher Chemical) dissolved in serum-free DMEM (freshly prepared prior to use) at final concentrations of 100, 200, or 300 μM for 3 h. Cells were then washed with PBS (×3), supplied with fresh serum-containing DMEM, and allowed to recover for 3 or 5 days before SA-β-gal staining (as described for etoposide). The regimen of 300 μM H_2_O_2_ for 3 h followed by 5-day recovery was designated optimal and was likewise validated in MV4-11 cells.

At ~80% confluency, plates containing MCF-7 cells were placed in dry-block heaters (Thermo Scientific) preset to 39 or 41 °C for 0.5, 1, or 2 h. After heat shock, plates were immediately returned to the incubator for 72 h. SA-β-gal staining proceeded as above. A 1-h exposure at 41 °C was most effective and was subsequently applied to both MCF-7 and MV4-11 cells to induce senescence.

# 17. **Cell viability assay**

Tumor cells were plated in 96-well plates with 6.0 × 10^3^ cells in each well and subsequently incubated overnight in a moist atmosphere of 5% CO_2_ and 37 °C. Then, different concentrations of test compounds or vehicles were added to triplicate wells. After incubation for an additional 72 h, 10 µL CCK-8 solution (Dojindo Molecular Technologies, catalog no. CK04-11) was added to each well, then the plates were incubated for 3 h at 37 °C. The absorbance was read at 450 nm on a Microplate Reader. The values of IC_50_ were calculated by the Logit method (log(inhibitor) vs normalized response) with the GraphPad software.

# 18. **Apoptosis assay**

Cells were placed in 6-well transparent plates and then treated with test compounds and vehicle in a moist atmosphere of 5% CO_2_ at 37 °C for 24 h. After that, the cells were washed twice with cold Cell staining Buffer (BioLegend, catalog no. 420201-BL) twice, and then were resuspended in Binding buffer (BioLegend, catalog no. 640914). 5 μL of FITC Annexin V (BioLegend, catalog no. 640914) and 10 μL of Propidium Iodide Solution (BioLegend, catalog no. 640914) were added to the resuspended cell solution, which was then incubated for 15 min at room temperature in the dark. Analysis of stained cells was performed by a flow cytometer (BD FACS Canto II).

# 19. **GLB1 siRNA knockdown**

A549 and senescent A549 (s-A549) cells were seeded in culture plates and transfected at approximately 60–70% confluence with a GLB1-targeting siRNA (UAUGUUCAGGGUAGAAUCCCU, GGAUUCUACCCUGAACAUACC) or a non-targeting control siRNA at a final concentration of 0.1 nM, using Lipofectamine 3000 (L3000001). At 72 h after transfection, GLB1 knockdown efficiency was evaluated by RT-qPCR and immunoblotting.

# 20. **PDOs culture**

Fresh lung tumor tissues were obtained from patients undergoing surgical resection after written informed consent was obtained. Tumor specimens were transported on ice in Hank's balanced salt solution (HBSS) supplemented with antibiotics and processed within 2 h after resection.

Tissues were washed three times with cold HBSS, minced into approximately 1-mm^3^ fragments, and enzymatically digested in DMEM/F12 containing 1 mg mL−1 collagenase/dispase and 0.001% DNase I at 37 °C for 2 h with intermittent agitation. The resulting cell suspension was triturated, passed through a 70-μm cell strainer, and centrifuged for 3 min. The cell pellet was resuspended in growth factor-reduced Matrigel and seeded as domes in pre-warmed culture plates. After Matrigel polymerization at 37 °C for 10 min, organoids were overlaid with organoid culture medium.

The culture medium consisted of serum-free DMEM/F12 supplemented with 20 ng/mL^−1^ basic fibroblast growth factor, 50 ng/mL^−1^ human epidermal growth factor, N2 supplement, B27 supplement, 10 μM Y-27632, and 1% penicillin-streptomycin. PDOs were maintained at 37 °C in a humidified incubator with 5% CO2, and the medium was changed every 2-3 days.

PDOs were passaged every week when organoids reached approximately 100–120 μm in diameter. For passaging, Matrigel domes were disrupted in cold DPBS, and organoids were collected by centrifugation. Organoids were then dissociated using TrypLE Express at 37 °C for 10 min, mixed with fresh Matrigel, and reseeded at a split ratio of 1:3–1:4.

# 21. **Calcein-AM/PI Live–Dead Staining of PDOs**

PDOs were established from tumor specimens obtained from five independent lung cancer patient donors. For each donor-derived PDO model, organoids were cultured, pooled, and evenly allocated into five wells, with approximately 50–55 organoids per well at the start of treatment. The five wells were assigned to vehicle control, Light only, LS-D only, LS-D plus Light, and Dox conditions.

Photoactivation was performed using a Kessil PR160-390 LED light source (390 nm). The LED was positioned perpendicular to and 30 cm above the PDO sample plane, and the samples were irradiated for 5 min. The estimated irradiance at 30 cm was 7.84 mW/cm^2^, corresponding to an estimated total light dose of 2.35 J/cm^2^. The light-only and LS-D plus light groups were irradiated under identical conditions.

Following the indicated treatments, PDOs were gently washed once with warm phosphate-buffered saline and incubated in phenol-red-free DMEM/F12 containing 2 μM Calcein-AM and 1 μg mL^-1^ propidium iodide (PI) for 30 min at 37 °C in the dark. After staining, the dye-containing medium was replaced with fresh culture medium, and images were acquired immediately using a high-content imaging system under identical exposure and acquisition settings for all experimental groups.

Calcein-AM positive regions were considered viable, whereas PI-positive regions were considered dead or membrane-compromised. Images were analyzed using ImageJ. The fraction was calculated as: Viable area (Calcein-AM area)/Total area (Calcein-AM area + PI area). Measurements from all organoids within each well were therefore aggregated at the well level to generate a single quantitative value for each donor–treatment pair. Thus, each donor contributed one biological replicate per treatment condition, rather than individual organoids being treated as independent replicates. Because all five treatment conditions were evaluated within each donor, statistical comparisons were performed using one-way repeated-measures ANOVA, with donor serving as the matched biological unit.

# 22. **^1^H and ^13^C NMR spectra**

^1^H and ^13^C NMR of LS-C

^1^H and ^13^C NMR of LS-D

^1^H and ^13^C NMR of LS-A

# 23. **High-resolution mass spectrometry (HRMS)**

HRMS of **LS-C**

HRMS find the right EM+1: 474.1392.

HRMS of **LS-D**

HRMS find the right EM+18: 1008.3243.

HRMS of **LS-A**

HRMS find the right EM+1: 1433.5024, and EM/2 + 1: 717.2561.
